# High-throughput screening identifies a critical role of the SPOP–PABPC1 axis in lung adenocarcinoma progression

**DOI:** 10.1073/pnas.2602470123

**Published:** 2026-06-10

**Authors:** Jiahui Zhang, Ran Liu, Xue Han, Yutong Jiao, Yun Peng, Zizhang Zhou, Yanran Deng, Lianggeng Gong

**Affiliations:** ^a^ Department of Radiology, The Second Affiliated Hospital, Jiangxi Medical College, Nanchang University, Nanchang 330006, China; ^b^ Department of Thoracic Surgery, the First Affiliated Hospital, Jiangxi Medical College, Nanchang University, Nanchang 330006, China; ^c^ Key Laboratory of Biodiversity Conservation and Bioresource Utilization of Jiangxi Province, College of Life Sciences, Jiangxi Normal University, Nanchang 330006, China; ^d^ Queen Mary College, Nanchang University, Nanchang 330006, China; ^e^ College of Life Sciences, Shandong Agricultural University, Tai’an 271018, China

**Keywords:** LUAD, SPOP, HAUSP, PABPC1, nondegradative ubiquitination

## Abstract

Lung adenocarcinoma (LUAD) is the most common and deadly subtype of lung cancer, with limited therapeutic options and poor prognosis. Ubiquitination is an important posttranslational modification (PTM) that controls protein turnover, localization and interaction. The dysregulated ubiquitination machinery is a hallmark of cancer, contributing to tumor development. Identifying key E3 ubiquitin ligases and deubiquitinases in LUAD could lead to new biomarkers and treatments. Through RNA sequencing, bioinformatics, and clinical studies, SPOP has been identified as a promising E3 ligase target in LUAD. SPOP is downregulated in LUAD samples and associated with poorer patient outcomes. Functional analyses demonstrate that SPOP suppresses LUAD cell migration, proliferation, and in vivo tumor growth. Mechanistically, SPOP targets poly(A)-binding protein cytoplasmic 1 (PABPC1) for nonproteolytic ubiquitination, leading to its nuclear retention and inhibition of global protein synthesis, thereby suppressing the oncogenic effects of PABPC1. Furthermore, the deubiquitinase HAUSP is able to counteract SPOP-mediated ubiquitination of PABPC1, enhancing its oncogenic role in LUAD cells. Collectively, our findings reveal a SPOP/HAUSP-PABPC1 regulatory axis in LUAD, where SPOP and HAUSP oppositely modulate PABPC1 ubiquitination to control LUAD cell proliferation and migration. This axis represents a promising therapeutic target in LUAD.

## Significance

Lung adenocarcinoma (LUAD) is a lethal subtype of lung cancer with limited therapeutic options. Through an integrated screening combining bioinformatics, transcriptomics, and clinical prognostic analysis, we have identified the E3 ubiquitin ligase SPOP as a key tumor suppressor in LUAD. Mechanistically, SPOP antagonizes the oncoprotein PABPC1 by catalyzing its nondegradative K63-linked poly-ubiquitination, leading to PABPC1 nuclear retention and inhibition of global protein synthesis, consequently suppressing tumor progression. Conversely, the deubiquitinase HAUSP reverses this modification, fostering cancer development. This study not only establishes the SPOP/HAUSP-PABPC1 axis as a crucial regulator of protein synthesis in LUAD but also provides a generalizable screening framework for identifying functional ubiquitin ligases in cancer, thereby facilitating the discovery of additional therapeutic targets.

Under physiological conditions, proteins undergo various posttranslational modifications (PTMs), including ubiquitination, to regulate their localization and stability (1). Eukaryotic cells execute ubiquitination through a three-step catalytic cascade involving ubiquitin-activating enzymes (E1s), ubiquitin-conjugating enzymes (E2s), and ubiquitin ligases (E3s) (2). E3s are regarded as the most influential among these enzymes owing to their ability to select, bind, and recruit substrates for ubiquitination (3). Similar to other PTMs, ubiquitination is a reversible process due to the functions of deubiquitinases (DUBs) (4). Ubiquitination is an important mechanism for eliminating misfolded or unwanted proteins in cells, and its dysfunction is a hallmark of tumors. Tumor cells frequently exhibit abnormal expression of E3s, leading to degradation of tumor suppressors or stabilization of oncoproteins (5). Many E3s are being considered as viable targets for cancer therapy (6–8). Therefore, a systematic screen of abnormally expressed E3s in tumor tissues can deepen the understanding of tumorigenesis and offer insights for potential drug targets.

The ubiquitin–proteasome system (UPS) is responsible for the turnover of most cellular proteins, making it a promising therapeutic strategy for cancer treatment (9, 10). The central regulatory mechanism of UPS relies on the balance between ubiquitination and deubiquitination, which is controlled by E3s and DUBs. Speckle-type POZ protein (SPOP) functions as a substrate-binding adaptor within the Cullin3/RING (CUL3) E3 ubiquitin ligase complex (11). As a cardinal member of the MATH-BTB family, SPOP contains an N-terminal MATH domain for substrate recognition (12), and a C-terminal BTB domain for binding CUL3 (11). This structural architecture enables SPOP to act as a molecular adaptor that specifically recruits substrates to the CUL3 complex, thereby catalyzing their ubiquitination (13). SPOP-mediated ubiquitination can be classified into degradative and nondegradative types (14). Emerging evidence suggests that SPOP plays crucial roles in the initiation and progression of various cancers. In prostate cancer, SPOP suppresses tumorigenesis by ubiquitinating and degrading oncoproteins, including SRC-3 and c-MYC (15, 16). Knocking down SPOP results in the stabilization of GLI2 and GLI3, the key effectors of the Hh pathway, thereby leading to the onset of several types of cancer (17). In addition, our previous study has demonstrated that SPOP promotes proteasomal degradation of the tumor suppressor IRF2BP2 to repress the progression of HCC (18). However, considerable controversy persists regarding the role of SPOP in lung cancer (19, 20), which underscores the imperative to elucidate its precise biological functions. Therefore, resolving these discrepancies is essential for gaining a definitive understanding of lung cancer pathogenesis.

RNA-binding proteins (RBPs) are pivotal posttranscriptional regulators that orchestrate key processes, including RNA splicing, localization, and translation, to coordinate complex gene expression networks (21). PABPC1, a crucial RBP, serves as a key controller of mRNA fate in the cytoplasm and plays a role in various physiological and pathological processes (22). By binding specifically to the poly(A) tail at the 3’ end of mRNA, PABPC1 regulates protein translation initiation and mRNA decay (23). Increasing evidence suggests a strong correlation between cancer development and dysregulation of PABPC1 expression (23). The increased levels of PABPC1 in various solid tumors, such as hepatocellular carcinoma (HCC), ovarian cancer, NSCLC, and gastric cancer, suggest its oncogenic properties (24–27). In HCC, PABPC1 facilitates tumor cell proliferation by enhancing the translation efficiency of *c-MYC* and *Cyclin D1* (28, 29). It also induces the epithelial–mesenchymal transition, leading to elevated growth and migration of ovarian cancer cells (25). Additionally, PABPC1 enhances gastric cancer cell survival and chemoresistance by facilitating *PIK3CB* mRNA translation to activate the JAK-STAT3 pathway (30). Besides, PABPC1 manages broader oncogenic effects by modulating key processes, including the Nrf2-mediated antioxidant response and PTEN-regulated growth control (31, 32). Despite extensive research showing the oncogenic role of PABPC1, the mechanisms regulating its activity remain elusive.

In this study, we provided bioinformatic and functional evidence identifying SPOP as a key tumor-suppressing E3 ligase in LUAD. SPOP inhibited LUAD cell migration, proliferation, and in vivo tumor growth. Importantly, we found that PABPC1 is a substrate of SPOP. Mechanistically, SPOP interacted with PABPC1 through its MATH domain to catalyze K63-linked ubiquitination of PABPC1 in a CUL3-dependent manner. This modification promoted the nuclear retention of PABPC1 without affecting its stability. Consequently, SPOP inhibited PABPC1-mediated global protein synthesis and counteracted the oncogenic functions of PABPC1. Furthermore, we found that deubiquitinase HAUSP bound PABPC1 to antagonize its ubiquitination, resulting in strengthened oncogenic effect of PABPC1. Thus, by regulating PABPC1-mediated global protein synthesis, the SPOP/HAUSP-PABPC1 axis plays an important role in LUAD progression.

## Results

### SPOP Downregulation in LUAD Is Correlated With Poor Prognosis.

**Fig. 1.**
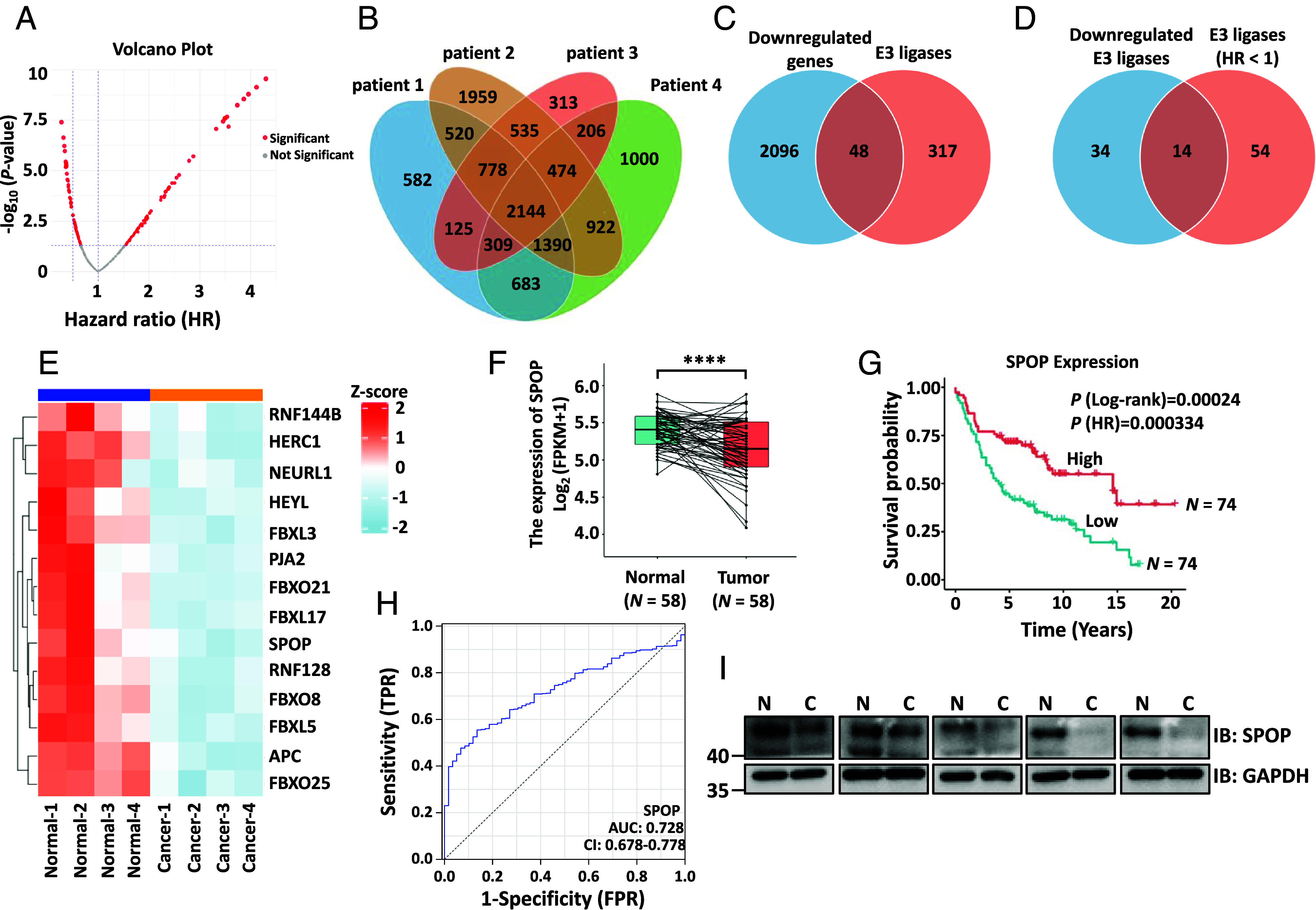
SPOP expression is decreased in LUAD samples and correlates with poor patient survival. (*A*) Volcano plot of the Cox regression analysis identifying prognostic E3 ligases associated with overall survival in the GEO database. (*B*) Venn diagram showing the overlap of downregulated genes from paired tumor-normal specimens across four independent LUAD patients. (*C*) Intersection of the shared downregulated genes (from *B*) with the E3 ligase gene set. (*D*) The downregulated E3 ligases (from *C*) were intersected with the favorable prognostic E3 ligases (HR < 1) (from *A*), identifying 14 candidate genes. (*E*) Heat map of the 14 candidate E3 ligases (from *D*) showing consistent downregulation in LUAD tumors compared to adjacent normal tissues. (*F*) LUAD tumors showed lower *SPOP* mRNA levels compared to adjacent normal tissues in the TCGA database. (*G*) Kaplan–Meier analysis showed that low SPOP expression is associated with significantly poorer overall survival in LUAD patients. (*H*) ROC curve analysis of SPOP expression for discriminating LUAD tumors from normal tissues in the TCGA dataset. (*I*) SPOP protein levels were reduced in LUAD cancer samples compared to matched adjacent normal tissues.

Dysregulated protein turnover is a hallmark of human cancers and represents a promising target for therapeutic interventions (9). One central mechanism involves proteasome-mediated degradation, which depends on ubiquitination, a PTM that is controlled by E3 ligases (33). Compelling evidence highlights the pivotal role of E3 ligases in tumorigenesis and the robust association with clinical outcomes (3). To identify key E3 ligases involved in lung adenocarcinoma (LUAD) development, we employed an integrated approach combining bioinformatics analysis with clinical sample validation. First, Cox regression analysis was conducted on E3 ligases that are differentially expressed in LUAD (*P* < 0.05) to evaluate their correlation with prognosis (Fig. 1*A*). Given that E3 ligases are primarily recognized for mediating oncoprotein degradation and frequently exerting antitumor functions, we prioritized those exhibiting a hazard ratio (HR) < 1, which yielded a list of 68 candidates. We further performed RNA sequencing (RNA-seq) analysis on paired tumor-normal samples from four LUAD patients. Intersection analysis across these patients identified 2144 overlapping downregulated genes (Fig. 1*B*). This downregulated gene set included 48 E3 ligases (Fig. 1*C*). By overlapping these 48 ligases with the 68 E3 ligases previously identified through Cox regression analysis, we obtained 14 candidate genes (Fig. 1*D*). A heatmap visualizing relative expression further confirmed the downregulation of these candidates across all LUAD samples (Fig. 1*E*). Due to the unclear significance of SPOP in LUAD pathogenesis, we selected it for further investigation.

To assess SPOP’s role in LUAD progression, we first analyzed its mRNA levels in TCGA database. Consistent with our RNA-seq results, *SPOP* transcriptional levels were downregulated in LUAD samples in comparison with adjacent normal tissues (Fig. 1*F*). An analysis of overall survival (OS) in LUAD patients from TCGA database demonstrated that low expression of SPOP was correlated with poorer clinical outcomes (Fig. 1*G*). Furthermore, receiver operating characteristic (ROC) analysis showed that SPOP levels exhibited diagnostic value in distinguishing LUAD from normal tissues, yielding an area under the curve (AUC) value of 0.728 (95 % CI, 0.678 to 0.778) (Fig. 1*H*). To validate reduced SPOP expression at the protein level, we collected five pairs of LUAD (cancer, C) and adjacent normal (normal, N) samples for immunoblotting. Consistently, LUAD samples exhibited lower SPOP protein levels compared to normal samples (Fig. 1*I*). Collectively, these findings suggest that the E3 ligase SPOP possibly plays an anti-tumor role in LUAD.

### SPOP Inhibits LUAD Cell Proliferation and Migration.

**Fig. 2.**
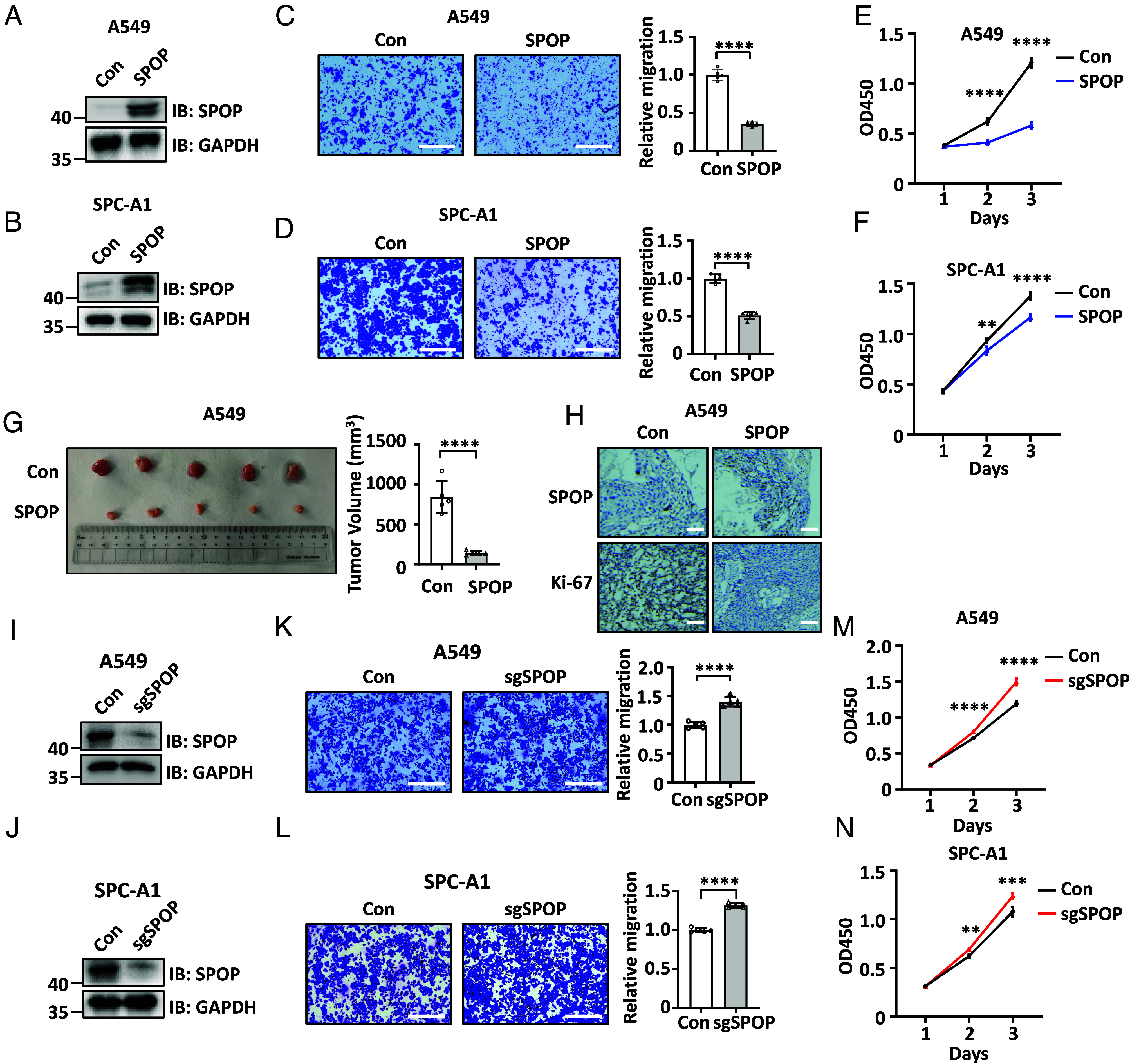
SPOP inhibits LUAD cell proliferation and migration. (*A* and *B*) Western blot detection of SPOP protein in A549 (*A*) and SPC-A1 (*B*) cells with stable SPOP overexpression. (*C* and *D*) Transwell assays demonstrated that overexpression of SPOP effectively suppressed the number of migrating A549 (*C*) and SPC-A1 (*D*) cells. Quantification analyses were shown on the *Right*. (Scale bar, 200 µm.) (*E* and *F*) CCK-8 proliferation assays in A549 (*E*) and SPC-A1 (*F*) cells revealed that overexpression of SPOP led to decreased cell viability. (*G*) The effect of SPOP overexpression was assessed in subcutaneous xenograft tumors formed by A549 cells. Quantification analysis of the final tumor volume was presented on the *Right* (*n* = 5). (*H*) Representative immunohistochemistry staining images showed elevated SPOP protein levels and reduced expression of the proliferation marker Ki-67 in xenograft tumor tissues. (Scale bar, 100 µm.) (*I* and *J*) Western blot detection of SPOP protein in A549 (*I*) and SPC-A1 (*J*) cells with sgSPOP. (*K* and *L*) Knockdown of SPOP promoted the migration of A549 (*K*) and SPC-A1 (*L*) cells. Quantification analyses were shown on the *Right*. (Scale bar, 200 µm.) (*M* and *N*) CCK-8 assays in A549 (*M*) and SPC-A1 (*N*) cells revealed that knockdown of SPOP led to increased cell viability. In all above, data were presented as means ± SD, **P < 0.01, ***P < 0.001, ****P < 0.0001.

To investigate the functional role of SPOP in LUAD, we selected A549 and SPC-A1 cell lines for subsequent experiments. Stable SPOP-overexpressing and empty vector control cell lines were successfully established in both A549 and SPC-A1 cells, as confirmed by western blotting (Fig. 2 *A* and *B*). Transwell assays showed that overexpression of SPOP led to a dramatic reduction in the migration of A549 and SPC-A1 cells (Fig. 2 *C* and *D*). To evaluate cell proliferation ability, we employed a series of in vitro assays. First, Cell Counting Kit-8 (CCK-8) assays revealed a marked decrease in cell viability in SPOP overexpressing cells compared to controls (Fig. 2 *E* and *F*). Next, colony formation assays showed that overexpression of SPOP reduced colony numbers (*SI Appendix,* Fig. S1 *A* and *B*), indicating a decrease in cell proliferative ability. Moreover, SPOP was able to suppress EdU incorporation in both A549 and SPC-A1 cells (*SI Appendix,* Fig. S1 *C* and *D*). To analyze SPOP’s role in vivo, we utilized a nude mouse xenograft model. In xenograft mice, overexpression of SPOP hampered tumor formation, leading to a reduction in tumor volume (Fig. 2*G* and *SI Appendix,* Fig S1*E*). Tumor tissue with SPOP overexpression consistently exhibited lower Ki-67 immunoreactivity, indicating a decreased proliferative capacity (Fig. 2*H* and *SI Appendix,* Fig S1*F*).

After demonstrating that overexpressing SPOP suppresses the proliferation and migration of LUAD cells, we proceeded to investigate the effects of reducing SPOP levels. Cas9-mediated gene disruption efficiently reduced the expression of endogenous SPOP in both A549 and SPC-A1 cells (Fig. 2 *I* and *J*). Compared with control cells (pLG3-*U6*), cells with decreased SPOP expression (pLG3-*U6*-sgSPOP) exhibited elevated capacities in migration (Fig. 2 *K* and *L*) and proliferation (Fig. 2 *M* and *N*). To validate these findings, we employed short hairpin RNA (shRNA) to silence the endogenous SPOP. Compared with control cells, shRNA treatment successfully suppressed the expression of SPOP in A549 and SPC-A1 cells (*SI Appendix,* Fig. S2 *A* and *B*). As expected, knockdown of SPOP indeed promoted cell migration (*SI Appendix,* Fig. S2 *C* and *D*) and proliferation (*SI Appendix,* Fig. S2 *E* and *F*). Furthermore, knockdown of SPOP facilitated colony formation in both A549 and SPC-A1 cells (*SI Appendix,* Fig. S2 *G* and *H*). Collectively, these results strongly suggest that SPOP depletion exerts a tumor-promoting role in LUAD cells.

### SPOP Requires Its Canonical Partner CUL3 to Exert Tumor-Suppressor Activity.

**Fig. 3.**
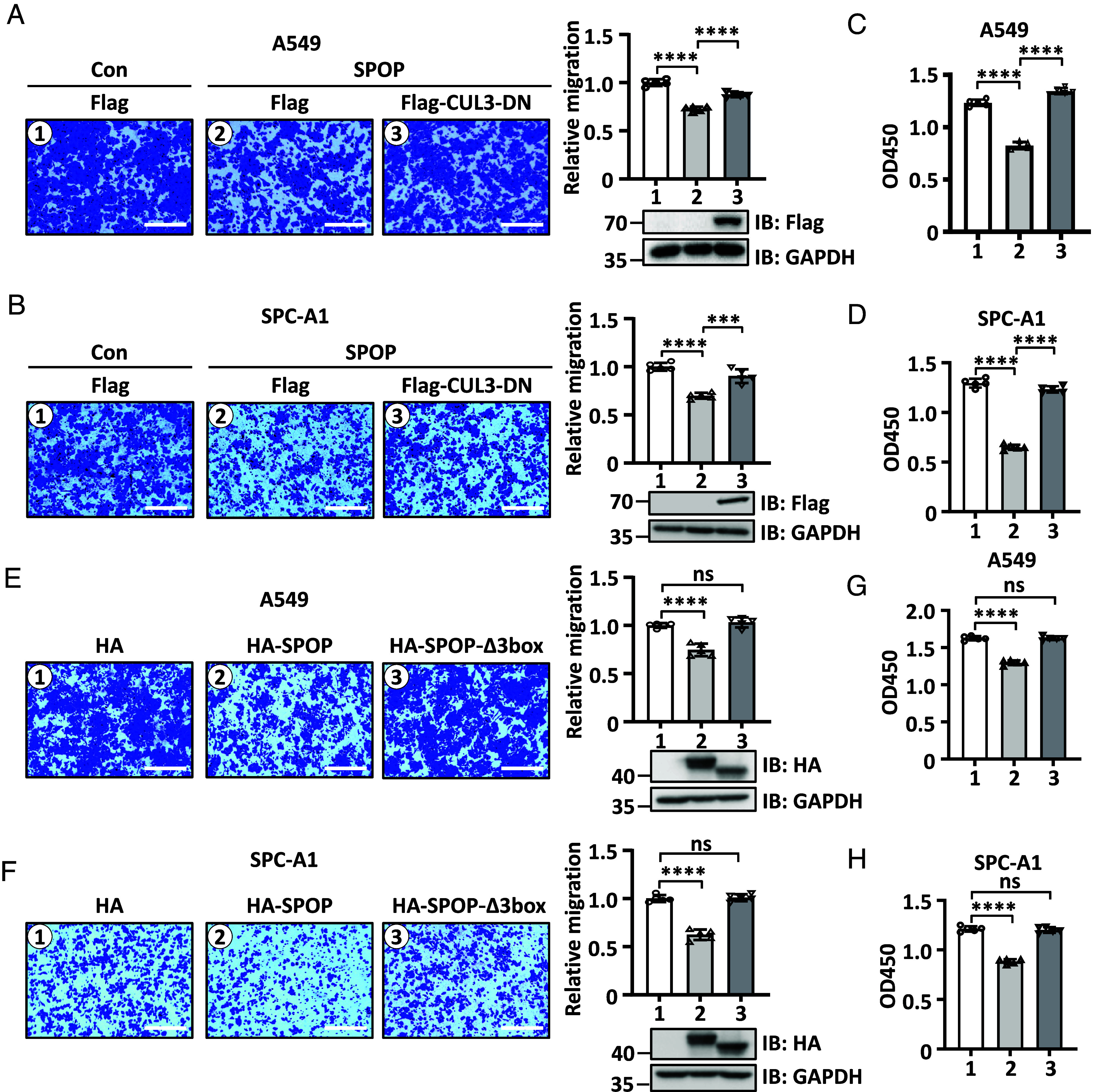
SPOP requires its canonical partner CUL3 to exert tumor-suppressor activity. (*A* and *B*) SPOP overexpression suppressed A549 (*A*) and SPC-A1 (*B*) cell migration, while co-overexpression of CUL3-DN abolished this inhibitory effect. The expression of constructs was assessed by using immunoblotting. Quantification analyses were shown on the *Right*. (*C* and *D*) SPOP-suppressed cell proliferation was restored by coexpression of CUL3-DN in A549 (*C*) and SPC-A1 (*D*) cells. (*E* and *F*) Wild-type SPOP inhibited A549 (*E*) and SPC-A1 (*F*) cell migration, whereas SPOP-Δ3box lost this capacity. The expression of constructs was examined using immunoblotting. Quantification analyses were shown on the *Right*. (*G* and *H*) Wild-type SPOP, but not SPOP-Δ3box was able to suppress A549 (*G*) and SPC-A1 (*H*) cell proliferation.

Given that SPOP typically functions as an E3 ubiquitin ligase in complex with its canonical partner CUL3 (34), we sought to examine whether SPOP’s anti-tumor role relies on CUL3. Within the CUL3-SPOP E3 ligase complex, CUL3 functions as a structural scaffold that recruits both the E2 ubiquitin-conjugating enzyme and the substrate adaptor SPOP. This assembly facilitates the transfer of ubiquitin from the E2 enzyme to specific substrates recognized by SPOP (35). We developed a truncated version of CUL3, CUL3-aa1-595, which lacks the C-terminus and cannot recruit E2s (36). This truncated version is referred to as CUL3-DN because of its dominant-negative role, as it competes with endogenous CUL3 for adaptor binding (18). In both A549 and SPC-A1 cells, transwell assays revealed that the inhibitory impact of SPOP on cell migration was effectively reversed by cotransfection with CUL3-DN (Fig. 3 *A* and *B*). Similarly, the reduction in cell viability induced by SPOP overexpression was rescued by coexpressing CUL3-DN (Fig. 3 *C* and *D*). Consistently, knockdown of CUL3 using a previously reported siRNA (37) effectively abolished the SPOP-mediated inhibition of cell migration and proliferation in both A549 and SPC-A1 cells (*SI Appendix,* Fig. S3 *A. B*, *D*, and *E*). The knockdown efficiencies of *CUL3* siRNA in both cells were confirmed by RT-qPCR (*SI Appendix,* Fig. S3 *C* and *F*).

To further validate that SPOP’s tumor-suppressive function is mediated by its E3 ligase activity, we employed a SPOP-Δ3box mutant lacking the CUL3-binding region, which disrupts the functional SPOP-CUL3 complex (33). In contrast to wild-type SPOP, the SPOP-Δ3box mutant exerted no inhibitory effect on cell migration in either A549 or SPC-A1 cells (Fig. 3 *E* and *F*). Furthermore, the proliferation of A549 and SPC-A1 cells was not affected by SPOP-Δ3box (Fig. 3 *G* and *H*). Overall, the tumor-suppressor activity of SPOP in LUAD cell lines is dependent on its E3 ligase activity.

### PABPC1 Is a Bona Fide Binding Partner of SPOP.

**Fig. 4.**
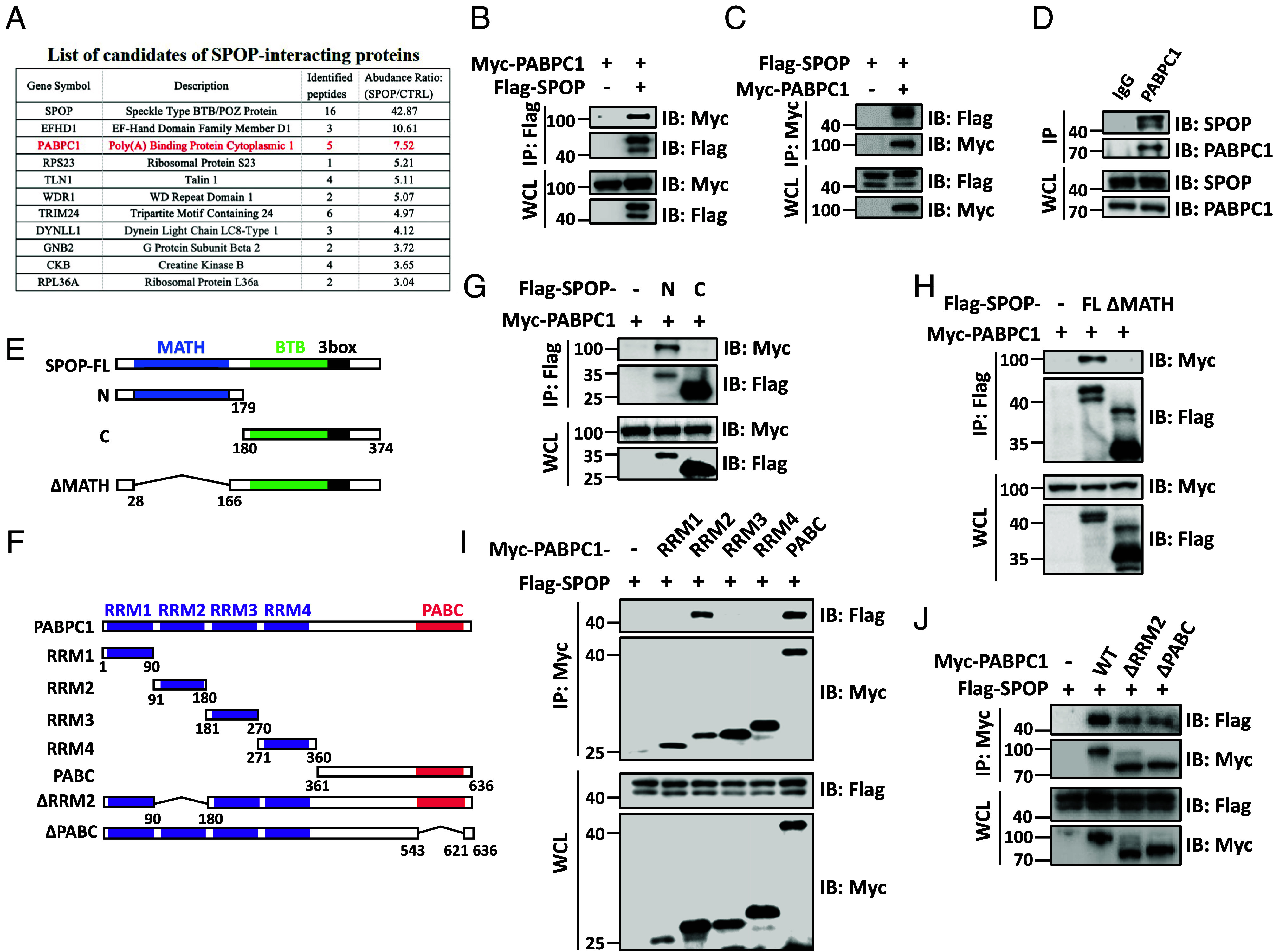
PABPC1 is a bona fide binding partner of SPOP. (*A*) Co-IP/MS analysis for screening of SPOP binding proteins in A549 cells. The number of peptides for each protein identified was listed. (*B*) Immunoblots of immunoprecipitates (IP, *Top* two panels) or whole cell lysates (WCL, *Bottom* two panels) from 293T cells expressing indicated plasmids. Of note, Flag-SPOP could pull down Myc-PABPC1. (*C*) Myc-PABPC1 was able to pull down Flag-SPOP. (*D*) Endogenous PABPC1 interacted with endogenous SPOP in A549 cells. (*E*) Schematic figures showed the domains in SPOP and the truncated mutants used in the subsequent studies. (*F*) Schematic figures display the domains in PABPC1, along with the truncated constructs utilized in the following Co-IP assay. (*G*) Flag-SPOP-N, but not Flag-SPOP-C interacted with Myc-PABPC1 in 293T cells. (*H*) Flag-SPOP-MATH, not Flag-SPOP-ΔMATH could pull down Myc-PABPC1 in 293T cells. (*I*) Flag-SPOP interacted with Myc-tagged RRM2-containing and PABC-containing regions in 293T cells. (*J*) Deletion of the RRM2 domain or the PABC domain of PABPC1 reduced its binding affinity for SPOP.

After showing SPOP plays tumor-suppressive role through its E3 ligase activity, we proceeded to identify the substrate responsible for this function. Using SPOP as the bait for coimmunoprecipitation coupled with mass spectrometry (Co-IP/MS) analysis in A549 cells, we identified PABPC1 as a potential interacting partner (Fig. 4*A*). Based on the oncogenic roles of PABPC1 in various types of tumor (23), it was selected for subsequent study. The Co-IP assays showed that Flag-SPOP indeed pulled down Myc-PABPC1 in HEK-293T cells (Fig. 4*B*). Reciprocally, Myc-PABPC1 also precipitated Flag-SPOP (Fig. 4*C*). Furthermore, PABPC1 was able to pull down SPOP in A549 cells (Fig. 4*D*), demonstrating their endogenous interaction.

To determine the domain of SPOP required for its interaction with PABPC1, we generated a series of truncation mutants for Co-IP assays (Fig. 4*E*). The Co-IP results showed that SPOP-N, rather than SPOP-C, pulled down PABPC1 (Fig. 4*F*). SPOP contains an N-terminal MATH domain, which is responsible for substrate recognition (18). To interrogate the requirement of the MATH domain in mediating the SPOP–PABPC1 interaction, we constructed a SPOP mutant with this domain deleted (SPOP-ΔMATH) (Fig. 4*E*). In contrast to full-length SPOP, the SPOP-ΔMATH mutant abrogated the interaction with PABPC1 (Fig. 4*H*), thereby establishing that the MATH domain within SPOP is both sufficient and necessary for mediating its interaction with PABPC1. PABPC1 is composed of four tandem RNA recognition motifs (RRMs) at its N terminus and a C-terminal PABC domain (also known as the MLLE domain) (Fig. 4*F*). In order to identify the SPOP-binding domain in PABPC1, various truncated PABPC1 mutants were cotransfected into 293T cells with SPOP. The Co-IP result revealed that both RRM2 and PABC domains of PABPC1 were able to pull down SPOP (Fig. 4*I*), whereas deletion of either domain markedly reduced their interaction (Fig. 4*J*), demonstrating that both domains are critical for the SPOP–PABPC1 association. Collectively, these findings identify PABPC1 as a bona fide interactor of SPOP.

### SPOP Mediates K63-Linked Polyubiquitination of PABPC1 and Promotes Its Nuclear Localization.

**Fig. 5.**
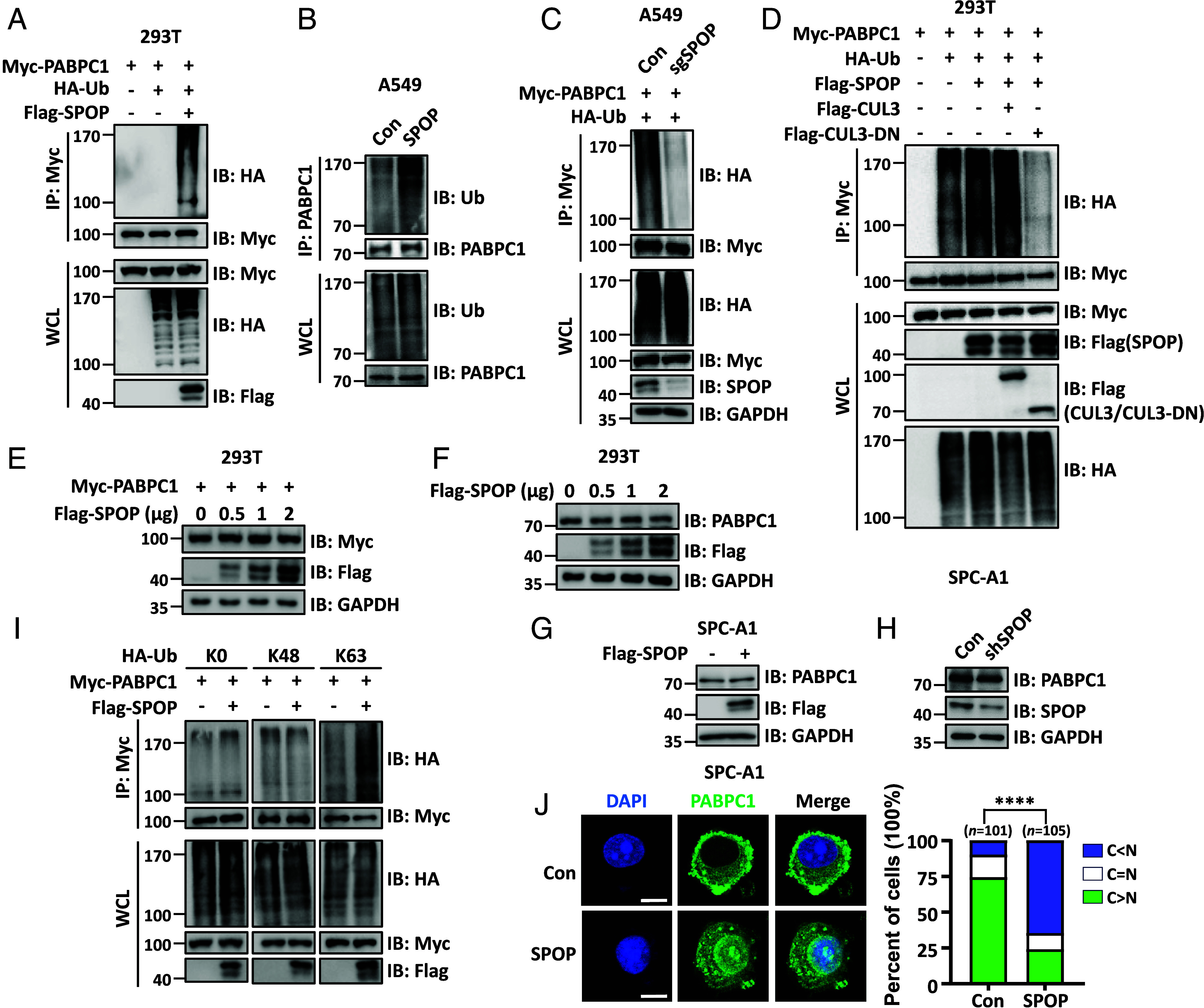
SPOP mediates K63-linked polyubiquitination of PABPC1 and promotes its nuclear localization. (*A*) Immunoblots of immunoprecipitates (IP, *Top* two panels) or whole cell lysates (WCL, *Bottom* three panels) from 293T cells transfected with the indicated plasmids. Of note, SPOP enhanced ubiquitination of PABPC1. (*B*) Overexpression of SPOP promoted the endogenous ubiquitination of PABPC1 in A549 cells. (*C*) Knockdown of SPOP reduced PABPC1 ubiquitination in A549 cells. (*D*) CUL3 enhanced, whereas CUL3-DN inhibited SPOP-mediated PABPC1 ubiquitination. (*E* and *F*) Increasing dose of Flag-SPOP did not alter the protein levels of exogenously expressed (*E*) and endogenous (*F*) PABPC1. (*G* and *H*) Endogenous PABPC1 levels were unaffected by either overexpression (*G*) or knockdown (*H*) of SPOP in SPC-A1 cells. (*I*) SPOP promoted K63-linked, but not K48-linked polyubiquitination of PABPC1. (*J*) Immunofluorescence assays revealed that SPOP overexpression induced the nuclear accumulation of PABPC1. Nuclei were stained with DAPI. Quantification of the fluorescence was shown on the *Right*. (Scale bar, 10 µm.) In all above, data were presented as means ± SD, ****P < 0.0001. In all above, data were presented as means ± SD, ***P < 0.001, ****P < 0.0001, ns, no significance. (Scale bar, 200 µm.)

Having demonstrated that PABPC1 is an interacting partner of SPOP, we next sought to investigate whether PABPC1 is a substrate of the SPOP-CUL3 E3 ligase. Co-IP assays showed that SPOP promoted the ubiquitination of PABPC1 in 293T cells (Fig. 5*A**)*. This finding was also confirmed in both A549 cells (Fig. 5*B* and *SI Appendix,* Fig. S4*A*) and SPC-A1 cells (*SI Appendix,* Fig. S4*B*). Conversely, knocking down endogenous SPOP in A549 cells reduced PABPC1 ubiquitination (Fig. 5*C*). In addition, the enhancement of SPOP-mediated PABPC1 ubiquitination was observed with coexpression of CUL3, while it was reduced with CUL3-DN (Fig. 5*D*), underscoring the crucial involvement of CUL3 in this process. These observations suggest that SPOP facilitates the ubiquitination of PABPC1 in a CUL3-dependent manner.

Since ubiquitination typically tags substrate proteins for proteasomal degradation (24), we sought to determine whether SPOP-mediated PABPC1 ubiquitination accelerates PABPC1 protein turnover. To this end, we transfected 293T cells with increasing amounts of a Flag-SPOP plasmid to assess its dose-dependent effect on PABPC1 protein levels. Notably, western blot analysis showed that Flag-SPOP had no detectable effect on the protein levels of either Myc-tagged or endogenous PABPC1 (Fig. 5 *E*- and *F*). Furthermore, neither overexpression nor knockdown of SPOP altered the protein levels of endogenous PABPC1 regardless of cycloheximide (CHX) treatment (Fig. 5 *G* and *H* and *SI Appendix,* Fig. S4 *C* and *D*), precluding a major role for SPOP in regulating PABPC1 turnover.

Since SPOP-mediated PABPC1 ubiquitination is nondegradative, we wanted to determine the types of this ubiquitination. We used the Ub-K0 mutant, in which all lysine residues were substituted with arginines to block polyubiquitin chain formation. Ub-K0 completely abolished SPOP-induced ubiquitination of PABPC1 (Fig. 5*I*), indicating that PABPC1 undergoes polyubiquitination. To define the chain linkage, we employed ubiquitin mutants that exclusively retain a single lysine. Co-IP analysis revealed that SPOP selectively catalyzed K63-linked polyubiquitination of PABPC1 (Fig. 5*I* and *SI Appendix,* Fig. S4*E*). Conversely, the Ub-K63R mutant, in which K63 is replaced by R, abolished SPOP-induced ubiquitination of PABPC1 (*SI Appendix,* Fig. S4*E*). Taken together, these results support that SPOP promotes K63-linked ubiquitination of PABPC1.

As K63-linked ubiquitination regulates protein trafficking (38), we examined the effect of SPOP on PABPC1 subcellular distribution. Immunofluorescence analysis revealed that SPOP facilitated the translocation of PABPC1 from the cytoplasm to the nucleus (Fig. 5*J*). We isolated cytoplasmic and nuclear PABPC1 and compared their K63-linked ubiquitination levels. Notably, nuclear PABPC1 exhibited increased K63-linked ubiquitination compared with its cytoplasmic counterpart (*SI Appendix,* Fig. S4*F*). Previous studies have demonstrated that the E3 ligase MKRN3 promotes K63-linked ubiquitination of PABPC1 at multiple lysine residues (39). Although both E3 ligases catalyze K63-linked ubiquitination of PABPC1, their outcomes were different. Unlike SPOP, MKRN3 only weakly facilitated the nuclear accumulation of PABPC1 (*SI Appendix,* Fig. S5*A*), suggesting that the two enzymes mediate ubiquitination at distinct sites. In support of this notion, although SPOP and MKRN3 enhanced K63-linked ubiquitination of PABPC1, a superimposed effect was observed when both were present, further confirming that they act on different sites (*SI Appendix,* Fig. S5*B*).

PABPC1 is a nucleoplasmic shuttling protein, and its nuclear import relies on interaction with Importin α-3 (encoded by *KPNA3*) (40). Co-IP assays revealed that KPNA3 substantially pulled down PABPC1, and this interaction was enhanced by SPOP overexpression (*SI Appendix,* Fig. S6*A*). In contrast, SPOP-Δ3box failed to promote the PABPC1–KPNA3 interaction (*SI Appendix,* Fig. S6*B*), underscoring the requirement of SPOP’s E3 ligase activity in facilitating this association. Furthermore, transfection of Ub-K63 increased the PABPC1–KPNA3 interaction (*SI Appendix,* Fig. S6*C*), phenocopying the effect of SPOP overexpression. Collectively, these findings indicate that SPOP-mediated K63-linked ubiquitination of PABPC1 strengthens its binding affinity for KPNA3, thereby promoting PABPC1 nuclear localization.

### SPOP Counteracts the Oncogenic Role of PABPC1 in LUAD Cells.

**Fig. 6.**
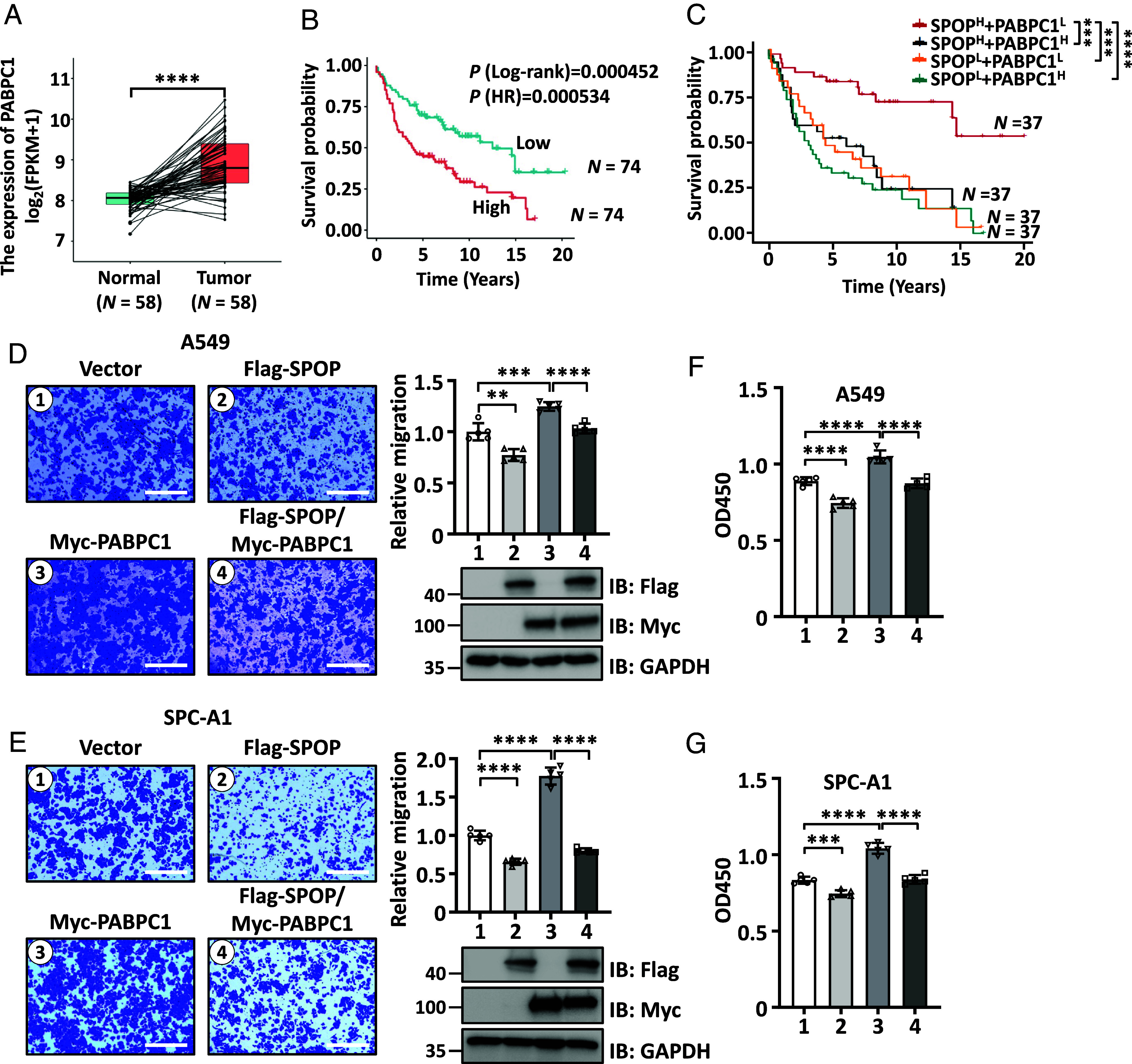
SPOP counteracts the oncogenic role of PABPC1 in LUAD cells. (*A*) Data from the GEO database showed elevated *PABPC1* mRNA levels in LUAD tumor tissues compared to normal tissues. (*B*) Kaplan–Meier analysis showed that high PABPC1 expression leads to poor overall survival in LUAD patients. (*C*) Kaplan–Meier curves show the overall survival of LUAD patients stratified into four groups based on high or low expression of SPOP and PABPC1. Patients with high SPOP and low PABPC1 levels exhibited the most prolonged survival compared to other groups. (*D* and *E*) Overexpression of PABPC1 restored SPOP-mediated inhibition of A549 (*D*) and SPC-A1 (*E*) cell migration. The expression of constructs was examined by immunoblotting. Quantification analyses were shown on the *Right*. (Scale bar, 200 µm.) (*F* and *G*) The inhibitory effect of SPOP on cell viability was attenuated by the coexpression of PABPC1 in A549 (*F*) and SPC-A1 (*G*) cells. In all above, data were presented as means ± SD, **P < 0.01, ***P < 0.001, ****P < 0.0001.

The observation that SPOP catalyzes nonproteolytic ubiquitination of PABPC1 prompted us to investigate their functional relationship. Analysis of transcriptomic data from the TCGA LUAD cohort revealed a significant upregulation of *PABPC1* mRNA in tumor tissues (Fig. 6*A*). A previous study has shown that high PABPC1 expression in hepatocellular carcinoma is associated with advanced TNM stage and poorer prognosis (24). Accordingly, survival analysis within the GSE30219 cohort demonstrated that elevated PABPC1 expression served as an independent predictor of poor patient outcomes (Fig. 6*B*). To assess the clinical relevance of the SPOP–PABPC1 axis, we analyzed their coexpression in relation to survival outcomes. The cohort was stratified into groups based on SPOP and PABPC1 levels. Patients with high SPOP/low PABPC1 had the best survival probability, in stark contrast to those with low SPOP/high PABPC1, who had the worst (Fig. 6*C*). These results establish SPOP as a suppressor of PABPC1 and underscore the clinical significance of this regulatory axis.

To determine whether SPOP suppresses the tumor-promoting functions of PABPC1, we coexpressed both proteins in LUAD cells and assessed their effects on migration and proliferation. In A549 cells, PABPC1 overexpression promoted migration, whereas SPOP coexpression completely abrogated this effect (Fig. 6*D*). This finding was confirmed in SPC-A1 cells (Fig. 6*E*). Similarly, the pro-proliferative effect of PABPC1 was fully rescued by SPOP in both cell lines (Fig. 6 *F* and *G*). Taken together, these results demonstrate that SPOP antagonizes the oncogenic phenotypes driven by PABPC1.

### SPOP and HAUSP Bidirectionally Regulate PABPC1 Ubiquitination.

**Fig. 7.**
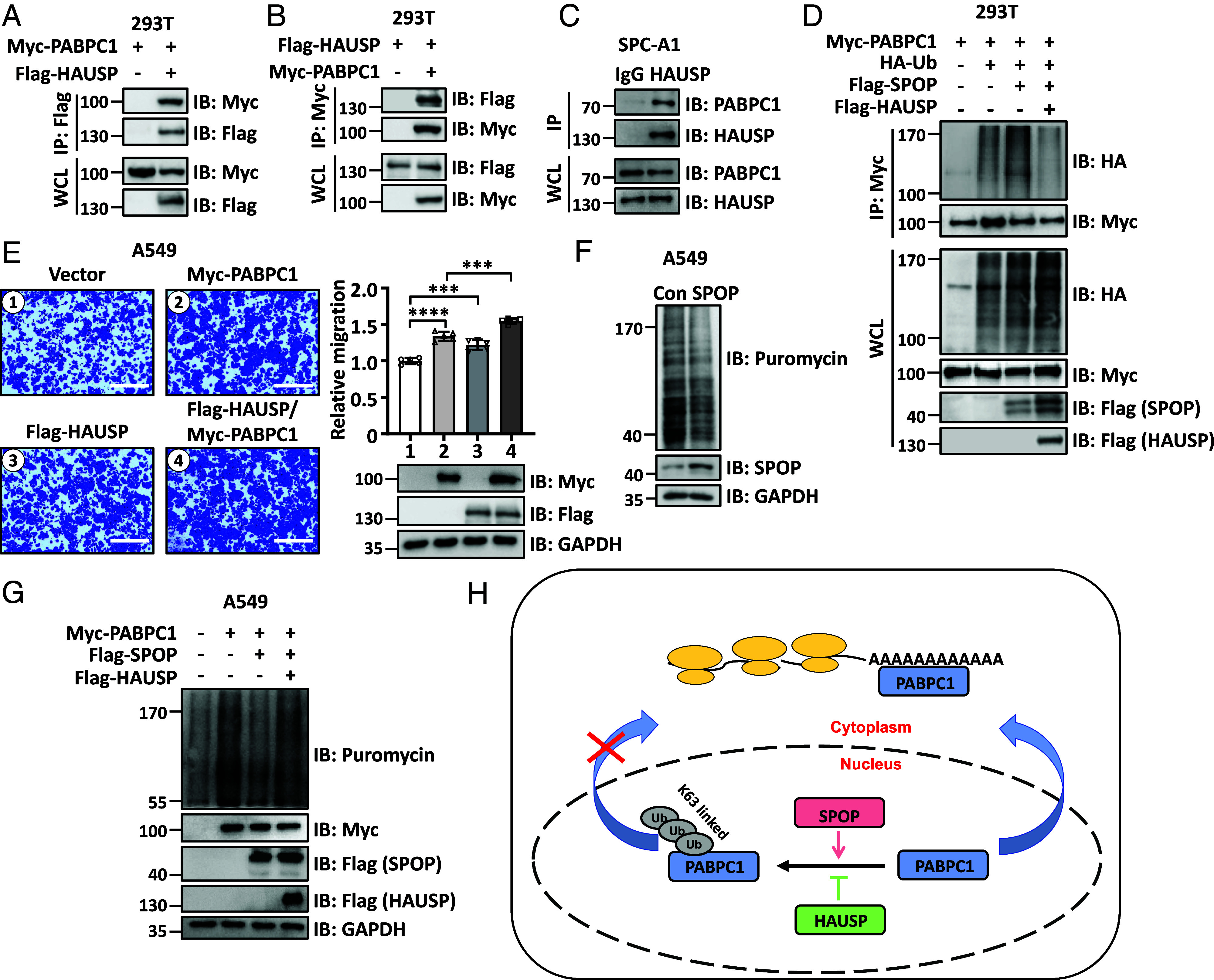
SPOP and HAUSP bidirectionally regulate PABPC1 ubiquitination. (*A*) Immunoblots of immunoprecipitates (IP, *Top* panels) or whole cell lysates (WCL, *Bottom* panels) from 293T cells expressing indicated plasmids. Of note, Flag-HAUSP could pull down Myc-PABPC1. (*B*) Myc-PABPC1 was able to pull down Flag-HAUSP. (*C*) Interaction between endogenous HAUSP and PABPC1 in SPC-A1 cells. (*D*) HAUSP reversed SPOP-induced ubiquitination of PABPC1. (*E*) HAUSP coexpression boosted PABPC1-induced A549 cell migration. The expression of constructs was examined by immunoblotting. Quantification analysis was shown on the *Right*. (Scale bar, 200 µm.) (*F*) SUnSET assay revealed that SPOP could inhibit global protein synthesis in A549 cells. (*G*) In A549 cells, SPOP suppressed PABPC1-induced global protein synthesis, and this effect was attenuated upon HAUSP coexpression. (*H*) A proposed model for SPOP and HAUSP regulating PABPC1. In all above, data were presented as means ± SD, ***P < 0.001, ****P < 0.0001.

Ubiquitination is a reversible protein modification due to the action of deubiquitinases (DUBs) (4). To identify DUBs responsible for PABPC1 deubiquitination, we performed Co-IP/MS analysis in A549 cells, leading to the finding of HAUSP as the only DUB among all PABPC1-interacting candidates. We validated this interaction by reciprocal Co-IP assays in 293T cells expressing Flag-HAUSP and Myc-PABPC1 (Fig. 7 *A* and *B*). Furthermore, endogenous HAUSP was able to interact with endogenous PABPC1 in SPC-A1 cells (Fig. 7*C*), confirming their association under physiological conditions. We next investigated whether HAUSP deubiquitinates PABPC1. Notably, HAUSP coexpression abolished SPOP-induced ubiquitination of PABPC1 (Fig. 7*D*). Furthermore, HAUSP also removed MKRN3-mediated ubiquitination of PABPC1 (*SI Appendix,* Fig. S7*A*), establishing PABPC1 as a bona fide substrate of HAUSP.

To assess the functional relevance of HAUSP-mediated PABPC1 deubiquitination in LUAD progression, we performed migration assays. In both A549 (Fig. 7*E*) and SPC-A1 cells (*SI Appendix,* Fig. S7*B*), HAUSP enhanced PABPC1-mediated cell migration. Since the nucleocytoplasmic shuttling of PABPC1 is known to influence its role in translation (41), we hypothesized that SPOP and HAUSP orchestrate the ubiquitination status of PABPC1 to ultimately affect protein synthesis. Using the nonradioactive SUnSET assay (42), we observed that overexpressing SPOP suppressed global protein synthesis (Fig. 7*F* and *SI Appendix,* Fig. S7*C*), whereas SPOP knockdown led to an increase in protein synthesis (*SI Appendix,* Fig. S7*D*). The stimulatory effect of PABPC1 on global translation was reversed by SPOP, whereas this reversal was abolished following HAUSP cotransfection in A549 (Fig. 7*G*) and SPC-A1 cells (*SI Appendix,* Fig. S7*E*). Collectively, these results establish that SPOP and HAUSP antagonistically regulate the oncogenic function of PABPC1 in LUAD by controlling its nondegradative ubiquitination and subsequent impact on mRNA translation.

## Discussion

LUAD, the most prevalent and lethal subtype of lung cancer, is characterized by high morbidity and mortality. Elucidating the molecular mechanisms underlying LUAD pathogenesis is therefore a pressing priority. Cellular proteomes are dynamically remodeled in response to physiological and environmental cues, with the timely removal and turnover of proteins being essential for maintaining proteostasis (43). The ubiquitin–proteasome system (UPS) is the primary mechanism for protein degradation, responsible for approximately 80% of proteome turnover. Its impairment leads to dysregulated protein homeostasis and accumulation of aberrant proteins, a recognized hallmark of tumorigenesis (44). E3 ligases are central to ubiquitination by conferring substrate specificity, making them promising therapeutic targets (38). Importantly, the human genome encodes over 600 E3 ligases, which means that studying their specific roles involves navigating substantial complexity (45). In this study, we performed a bioinformatics-driven high-throughput screen of E3 ligases by integrating their expression profiles and prognostic HR in clinical LUAD samples. From this analysis, SPOP was selected for further investigation based on its downregulated expression and association with favorable prognosis (HR < 1). Functional validation using both gain- and loss-of-function assays in LUAD cell lines and a nude mouse xenograft model established SPOP as a potent tumor suppressor. Mechanistically, SPOP interacts with PABPC1 to catalyze its nonproteolytic K63-linked polyubiquitination, which promotes the nuclear accumulation of PABPC1 (Fig. 7*H*). SPOP-mediated nuclear localization of PABPC1 attenuates global mRNA translation, thereby suppressing its oncogenic function. This ubiquitination is dynamically opposed by HAUSP, which removes ubiquitin chains from PABPC1 to restore its oncogenic activity (Fig. 7*H*). Overall, this study not only identifies that SPOP/HAUSP catalyzes reversible ubiquitination of PABPC1, but also establishes a generalizable, data-driven high-throughput screening strategy for identifying functional ubiquitin ligases that can be applied to other cancer types.

SPOP is frequently mutated across several cancer types, yet its functional consequences exhibit notable context-dependency (46, 47). In malignancies such as prostate and endometrial cancer, most SPOP mutations are loss-of-function, which impairs its ability to bind oncogenic substrates (48–50). In contrast, we previously identified a gain-of-function mutation, M35L, in liver cancer (18). This mutation reprograms SPOP from a tumor suppressor into an oncoprotein by enhancing its interaction with the tumor suppressor IRF2BP2 (18). Similarly, colorectal cancer-derived SPOP mutants, including F32L, S54T, and A61T, display strengthened affinity for LZTS2, further underscoring that distinct mutations differentially reshape SPOP activity (36). According to the cBioPortal database, LUAD samples exhibit an extremely low frequency of SPOP mutations (<1%), suggesting that mutations in this gene are unlikely to serve as a primary driver of LUAD tumorigenesis. Our study reveals that SPOP functions as a potent tumor suppressor in LUAD by broadly inhibiting global protein synthesis rather than targeting a specific pathway. This central role in translational control highlights the potential for treating LUAD by targeting SPOP. Recent studies have demonstrated that proteolysis-targeting chimera (PROTAC) degraders can recruit SPOP to its substrates, such as GLP and PD-L1, thereby accelerating their degradation (51, 52). It would therefore be fruitful to develop a PROTAC degrader that facilitates SPOP-mediated ubiquitination of PABPC1.

Over the past few decades, numerous oncogenic substrates of SPOP have been identified, including AR, GLI2, c-MYC, and TRIM24 (13, 53–55). On the other hand, SPOP also promotes the degradation of tumor suppressors such as PTEN and IRF2BP2 (18, 56). Thus, the pathological role of SPOP in distinct cancers is determined by the specific substrates it ubiquitinates. Here, we uncovered that oncoprotein PABPC1 is a substrate of SPOP in LUAD. Notably, rather than leading to proteasomal degradation, SPOP-mediated ubiquitination promoted the nuclear retention of PABPC1, thereby inhibiting PABPC1-induced protein translation. This finding implicates SPOP in a global protein translational control mechanism via repression of PABPC1, extending beyond its canonical role in posttranslational degradation. As elevated protein synthesis is a hallmark of proliferating cancer cells (57), targeting this process constitutes a promising therapeutic strategy. However, direct inhibition of the protein synthesis faces challenges due to its critical roles in numerous physiological processes. The SPOP/HAUSP-PABPC1 axis uncovered in this study offers an alternative therapeutic strategy. Targeting this axis may therefore enable the selective disruption of tumor-promoting translation in LUAD, potentially avoiding the systemic toxicity of broad protein synthesis inhibitors.

PABPC1 serves as a central regulator of mRNA fate, involves in diverse physiological and pathological processes (22). PABPC1 plays a critical role in regulating protein translation initiation and mRNA degradation by specifically binding to the poly(A) tail at the 3’ end of mRNA (58). Although primarily localized in the cytoplasm, PABPC1 functions as a nucleocytoplasmic shuttling protein and can translocate to the nucleus under specific conditions. For example, during viral infection, nuclear retention of PABPC1 has been shown to suppress host gene expression and restrict innate immune responses (59, 60). Moreover, a recent study in embryonic stem cells revealed that nuclear PABPC1 binds to (A)-rich circRNAs and restricts their nuclear export. Upon neuronal differentiation, reduced nuclear localization of PABPC1 enables the export of (A)-rich circRNAs (61). These findings underscore the tightly link between the subcellular localization of PABPC1 and its function.

## Materials and Methods

### Tumor and Tissue Samples.

Deidentified, snap-frozen tumor biopsies and matched normal samples were obtained from lung cancer patients at First Affiliated Hospital of Nanchang University. Written informed consent was obtained from all patients, and the study received approval from the Institutional Review Board of the First Affiliated Hospital, Jiangxi Medical College, Nanchang University [approval No. (2025) CDYFYYLK (09-013)]. All sample collections were conducted with institutional review board approval.

### Cell Lines, Transfection, and Infection.

The HEK-293T (293T) and A549 cell lines were obtained from the American Type Culture Collection (ATCC), while the SPC-A1 cell line was purchased from the China Center for Type Culture Collection (CCTCC). 293T and A549 cells were cultured in Dulbecco’s Modified Eagle Medium (Gibco), and SPC-A1 cells were maintained in RPMI-1640 medium (Gibco) supplemented with 10% fetal bovine serum (ExCell) at 37 °C in a 5% CO_2_ humidified atmosphere. Regular PCR-based assays were conducted to monitor *Mycoplasma* contamination, all of which yielded negative results. 293T cells were transfected via PEI (Polysciences), while A549 and SPC-A1 cells were transfected using Lipofectamine 3000 (Thermo Fisher Scientific) according to the manufacturer’s instructions. SPOP-knockdown LUAD cell lines were established using the CRISPR-Cas9 system. The pLG3-*U6*-sgSPOP plasmid was cotransfected with Cas9 into LUAD cells using Lipofectamine 3000, and the knockdown efficiency was confirmed by western blot.

For lentivirus infection, pLKO.1-*MOCK* shRNA, pLKO.1-*SPOP* shRNA, pLVX-IRES-puro, and pLVX-SPOP were cotransfected with the packaging plasmids psPAX2 and pMD2G into 293T cells using Lipofectamine 3000. Viral supernatants were harvested at 48 h after transfection and used to infect A549 and SPC-A1 cells in the presence of 10 μg/mL polybrene (Hanbio Biotechnology). After being selected with puromycin (Solarbio) for three consecutive passages, the infected cells were confirmed by western blot.

### DNA Constructs.

The full-length PABPC1, SPOP, HAUSP, MKRN3, CUL3, and Ub coding sequences were amplified via PCR using an A549 cDNA template, followed by subcloning into pcDNA3.1-Flag, pcDNA3.1-Myc, or pcDNA3.1-HA backbone vectors. Truncated fragments were engineered through inserting the indicated coding sequences into corresponding vectors. The point mutation constructs used in this study were generated on an HA-Ub backbone via PCR-based site-directed mutagenesis according to our previous study (62). The single guide RNA (sgRNA) sequence “CCT CCG GCA GAA ATG TCG AG” targeting SPOP was cloned into the pLG3-*U6* plasmid. The *SPOP* and *MOCK* short hairpin RNAs were inserted into vector PLKO.1-TRC. The target sequences used were as follows: *SPOP*-shRNA, 5’-GGT GCT ACA CAC AGA TCA AGG-3’; *MOCK*-shRNA, 5’-CAA CAA GAT GAA GAG CAC CAA-3’. *NC siRNA,* 5’-UUC UCC GAA CGU GUC ACG UdTdT-3’ (37); *CUL3* siRNA, 5’-GUC GUA GAC AGA GGC GCA AdTdT-3’ (37).

### RNA Extraction and RT-qPCR.

Total RNAs were extracted from A549 and SPC-A1 cells after transfection with TRIzol (Invitrogen) following standard protocols (63). The RNA was reverse transcribed using HiScript^®^ Q RT SuperMix with gDNA wiper (Vazyme) following the manufacturer’s instructions. The primer pairs used were as follows: *ACTIN*, 5’-CAT GTA CGT TGC TAT CCA GGC-3’ (forward) and 5’-TCC TTA ATG TCA CG CAC GAT-3’ (reverse); *CUL3*, 5’-CTC GAA GGA AGA TCA GTC TAT G-3’ (forward) and 5’-CTT CAT TAA TTC TAG CTT CTA C-3’ (reverse). Data are presented as means ± SD of values from at least three repeats. Real-time PCR was performed using ChamQ SYBR^®^ Color qPCR Master Mix (Vazyme) on ZY/VQ-100A (Yuanzan). Relative expressions of indicated genes were detected using the 2^−ΔΔCt^ method.

### Bioinformatics Analysis.

The expression of SPOP and PABPC1 in human LUAD tissues and paired adjacent normal tissues was analyzed using datasets from The Cancer Genome Atlas (TCGA). Gene expression profiles were downloaded from the Gene Expression Omnibus (GEO) database, and normalized using a log_2_ (FPKM+1) transformation. Statistical analysis, visualization of prognostic analysis, and grouping analysis were carried out using the *R* Studio software.

### Western Blotting and Immunofluorescence.

Cells were harvested for immunoprecipitation (IP) and immunoblotting (IB) assays as described previously (64). Antibodies used were as follows: rabbit anti-PABPC1 (Proteintech), rabbit anti-HAUSP (Proteintech), rabbit anti-SPOP (Proteintech), rabbit anti-Ubiquitin (Proteintech), rabbit anti-Puromycin (ABclonal), rabbit anti-GAPDH (Proteintech), mouse anti-Myc (Santa Cruz), mouse anti-Flag (Sigma), mouse anti-HA (Santa Cruz), goat anti-rabbit HRP (Abmax), and goat anti-mouse HRP (Abmax). For Surface Sensing of Translation (SUnSET) assays, cells were incubated with 10 µg/mL puromycin (Solarbio) for 10 min before harvesting, and then collected for western blot. The uncropped, full-length original western blots are provided in the Supplementary Information.

Cell-based immunofluorescence assays were performed as previously described (65). For EdU incorporation assays, cells were transfected with indicated plasmids and then plated on chamber slides. These cells were next incubated with 10 μM EdU (CellorLab) for 2 h before cell harvesting. The subsequent immunofluorescence steps were carried out following the previous protocol (65).

### Cell Counting Kit-8 (CCK-8) Assay.

A549 or SPC-A1 cells transfected with indicated plasmids were seeded into 96-well plates at a density of 2,000 cells/well in triplicate. 10 μL of CCK-8 (UElandy) was added to each well at 24, 48, and 72 h. After incubation for 2 h, OD values were measured at 450 nm using a microplate absorbance instrument (Bio-Rad).

### Transwell Assay.

After trypsinization, a suspension of 1 × 10^5^ cells in 300 μL of serum-free medium was placed into the upper chambers of transwell inserts (Corning), and 500 μL of medium containing 10% FBS was added to the lower chamber as chemoattractant. After incubation for 12 h or 24 h at 37 °C in 5% CO_2_, nonmigrating cells on the upper surface of the membrane were removed by scrubbing with cotton swabs. Then, the cells that had migrated to the bottom surface were fixed with 20% methanol for 20 min and stained with 0.1% crystal violet (Sangon Biotech). Finally, the stained cells were photographed and quantified under a microscope in 8 randomly chosen fields.

### Colony Formation Assay.

For colony formation assay, a single-cell suspension was seeded into 6-well plates with a density of 2,000 cells per well and cultured in complete medium for 2 wk. The resulting colonies were fixed with 4% formaldehyde for 20 min and stained with 0.1% crystal violet for 1 h. The number of colonies containing more than 50 cells was counted.

### Xenograft Experiment and Immunohistochemistry.

For the xenograft experiment, we randomly divided 6-wk-old BALB/c nude female mice into two groups (5 mice per group). To generate tumors, mice were injected subcutaneously in the left flank with 1×10^6^ A549 cells stably overexpressing SPOP or control cells transfected with empty vector. All mouse experiments were conducted following protocols approved by the Institutional Animal Care and Use Committee of The First Affiliated Hospital of Nanchang University. Mice were euthanized 4 wk after injection, and the resulting tumors were excised for volume measurement. Tumor volume was calculated using the following formula: width^2^×length/2. All animal procedures were approved by the First Affiliated Hospital of Nanchang University (approval No. CDYFY-IACUC-202509GR006).

Immunohistochemistry was performed on xenograft tumor tissues to evaluate the expression of SPOP and Ki-67. The sections were incubated overnight with primary antibodies, including rabbit anti-SPOP (1:200) and rabbit anti-Ki-67 (1:500; ProteinTech). Following washes, a secondary anti-rabbit IgG antibody was applied. Mayer’s hematoxylin was used for counterstaining, and representative images were captured using a microscope equipped with corresponding software.

### Statistical Analysis.

Statistical analyses were performed using GraphPad Prism software (GraphPad Software Inc., La Jolla, CA) with a two-tailed unpaired Student’s *t*-test. All data shown in this study were from at least three independent replicates and were presented as means ± SD. *P* values < 0.05 were considered statistically significant (ns, no significance, *P < 0.05, **P < 0.01, ***P < 0.001 and ****P < 0.0001).

## Supplementary Material

Appendix 01 (PDF)

## Data Availability

All study data are included in the article and/or *SI Appendix*.
