# Supplementary material for: High-throughput screening identifies a critical role of the SPOP–PABPC1 axis in lung adenocarcinoma progression
Source: Proc Natl Acad Sci U S A. 2026 Jun 10;123(24):e2602470123. doi: 10.1073/pnas.2602470123 (PMC13273242; doi:10.1073/pnas.2602470123)
Supplement: Supplementary file 1 — Appendix 01 (PDF) [file pnas.2602470123.sapp.pdf]

High-throughput screening identifies a critical role of the SPOP-PABPC1 axis in lung adenocarcinoma progression

Jiahui Zhang *et al.*

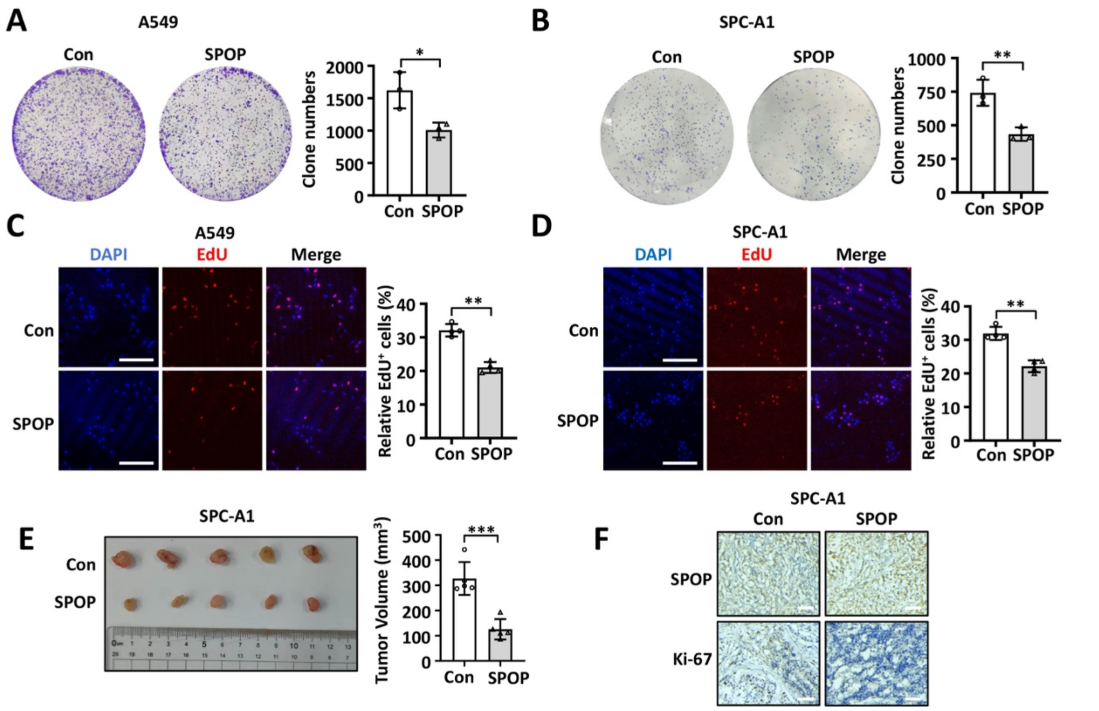

Figure S1. Overexpression of SPOP suppresses LUAD cell migration and proliferation

(**A-B**) Overexpression of SPOP inhibited colony formation in both A549 (A) and SPC-A1 (B) cells.

(**C-D**) EdU incorporation assays revealed that SPOP inhibited A549 (C) and SPC-A1 (D) cell proliferation. Quantification analyses were shown on the right. Scale bars, 200 μm.

(**E**) The effect of SPOP overexpression was assessed in subcutaneous xenograft tumors formed by SPC-A1 cells. Quantification analysis of the final tumor volume was presented on the right (n = 5).

(**F**) Representative immunohistochemistry staining images showed elevated SPOP protein levels and reduced expression of the proliferation marker Ki-67 in xenograft tumor tissues. Scale bars, 100 μm.

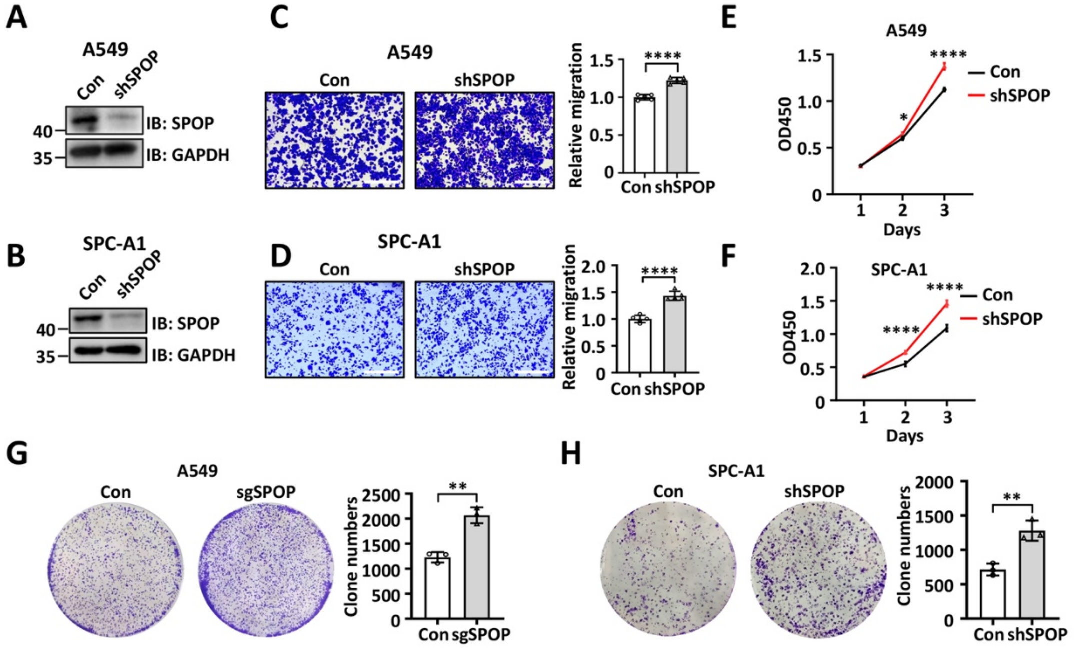

Figure S2. Knockdown of SPOP promotes the migration and proliferation of LUAD cells

(**A-B**) Western blot detection of SPOP protein in A549 (A) and SPC-A1 (B) cells with SPOP knockdown.

(**C-D**) Transwell assays demonstrated that knockdown of SPOP enhanced the number of migrating A549 (C) and SPC-A1 (D) cells. Quantification analyses were shown on the right. Scale bars, 200 μm.

(**E-F**) CCK-8 proliferation assays in A549 (E) and SPC-A1 (F) cells revealed that knockdown of SPOP led to increased cell viability.

(**G-H**) Knockdown of SPOP promoted colony formation in both A549 (G) and SPC-A1 (H) cells.

In all above, data were presented as means ± SD, $*P < 0.05$, $**P < 0.01$, $****P < 0.0001$.

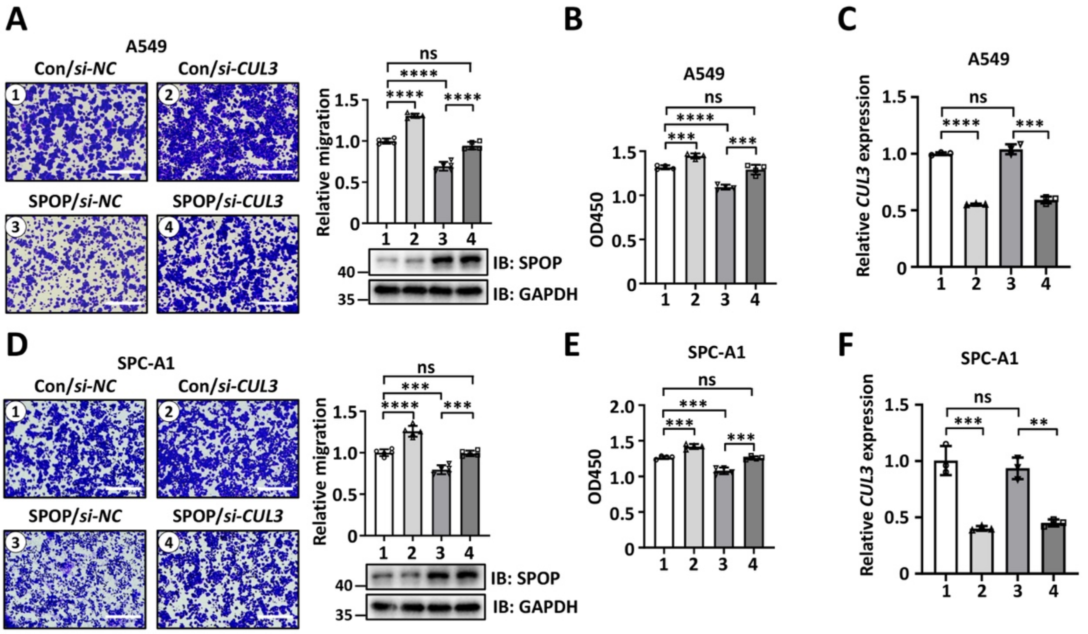

Figure S3. Knockdown of CUL3 abolishes the SPOP-mediated inhibition of cell migration and proliferation

(A-C) In A549 cells, SPOP-mediated inhibition of migration and proliferation was abrogated by CUL3 knockdown. The efficiency of CUL3 siRNA was confirmed via RT-qPCR (C).

(D-F) Knockdown of CUL3 reversed the SPOP-induced suppression of migration and proliferation in SPC-A1 cells. The knockdown efficiency of CUL3 siRNA was verified by RT-qPCR (F).

In all above, data were presented as means ± SD, $^{**}P < 0.01$, $^{***}P < 0.001$, $^{****}P < 0.0001$, ns, no significance.

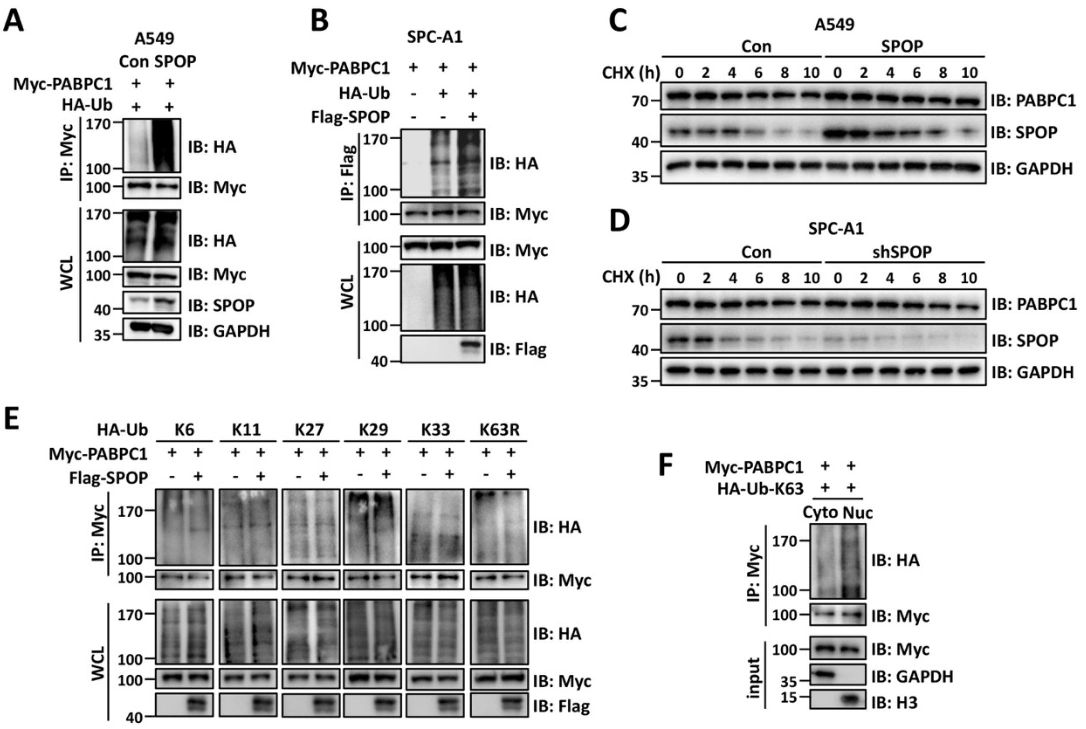

Figure S4. SPOP promotes non-degradative K63-linked ubiquitination of PABPC1

(**A-B**) Overexpression of SPOP promoted ubiquitination of PABPC1 in A549 (A) and SPC-A1 (B) cells.

(**C-D**) Following treatment with CHX (20 μg/mL), the degradation rate of endogenous PABPC1 remained unchanged upon either SPOP overexpression in A549 cells (C) or knockdown in SPC-A1 cells (D).

(E) SPOP catalyzed K63-linked ubiquitination of PABPC1.

(**F**) Nuclear PABPC1 displayed pronounced K63-linked ubiquitination.

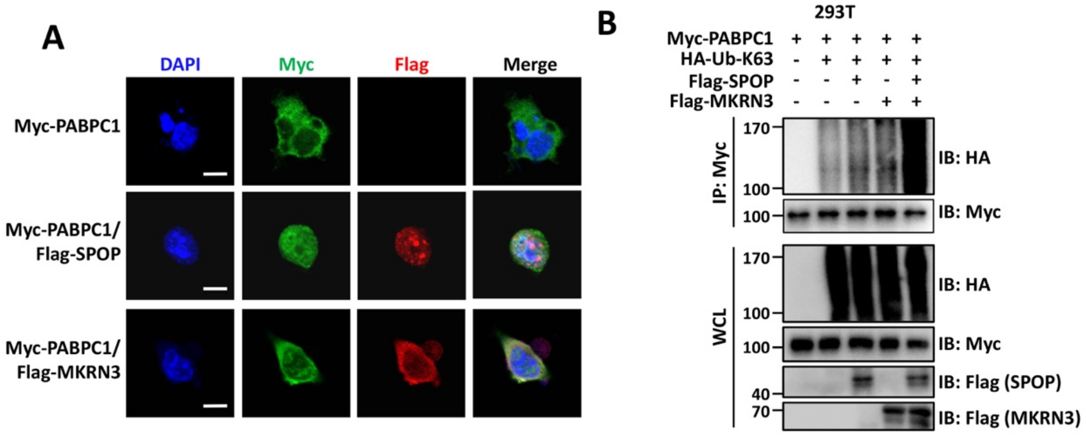

Figure S5. SPOP and MKRN3 catalyze PABPC1 ubiquitination at distinct lysine residues

(**A**) The subcellular localization of PABPC1 with SPOP or MKRN3 co-expression.

(**B**) SPOP and MKRN3 exerted an additive effect on the K63-linked ubiquitination of PABPC1.

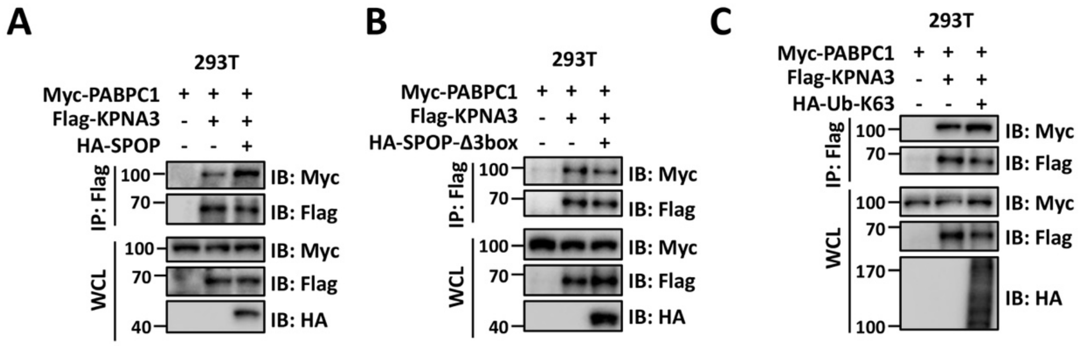

Figure S6. SPOP promotes the interaction between PABPC1 and KPNA3

(**A**) SPOP facilitated the association of PABPC1 with KPNA3.

(**B**) SPOP-Δ3box did not affect the PABPC1-KPNA3 interaction.

(**C**) Transfection of Ub-K63 increased the interaction between PABPC1 and KPNA3.

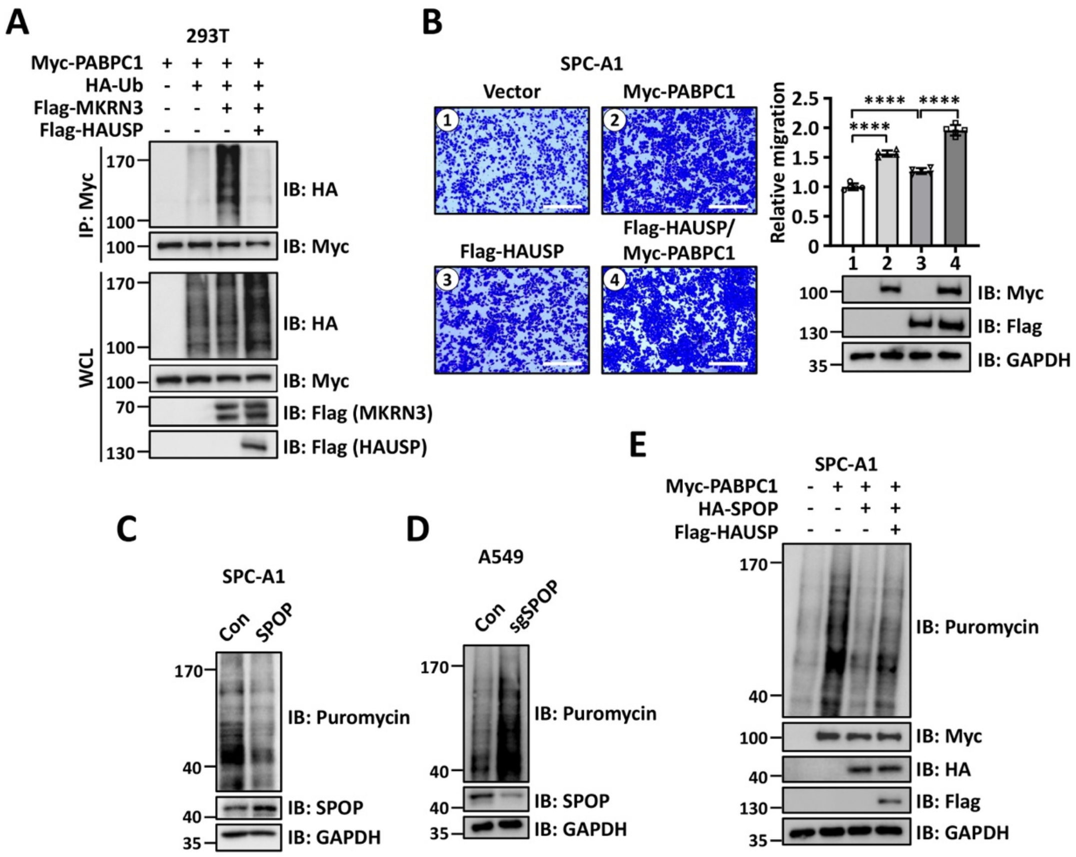

Figure S7. SPOP and HAUSP bidirectionally modulate PABPC1 activity

(**A**) HAUSP antagonized MKRN3-mediated ubiquitination of PABPC1.

(**B**) HAUSP enhanced PABPC1-mediated cell migration in SPC-A1 cells. Scale bars, 200 μm.

(**C**) Overexpression of SPOP led to a decrease in protein synthesis in SPC-A1 cells.

(**D**) SPOP knockdown promoted protein synthesis in A549 cells.

(**E**) HAUSP rescued SPOP-mediated inhibition of PABPC1-driven protein synthesis.

In all above, data were presented as means ± SD, ****$P < 0.0001$.

Original WB figures

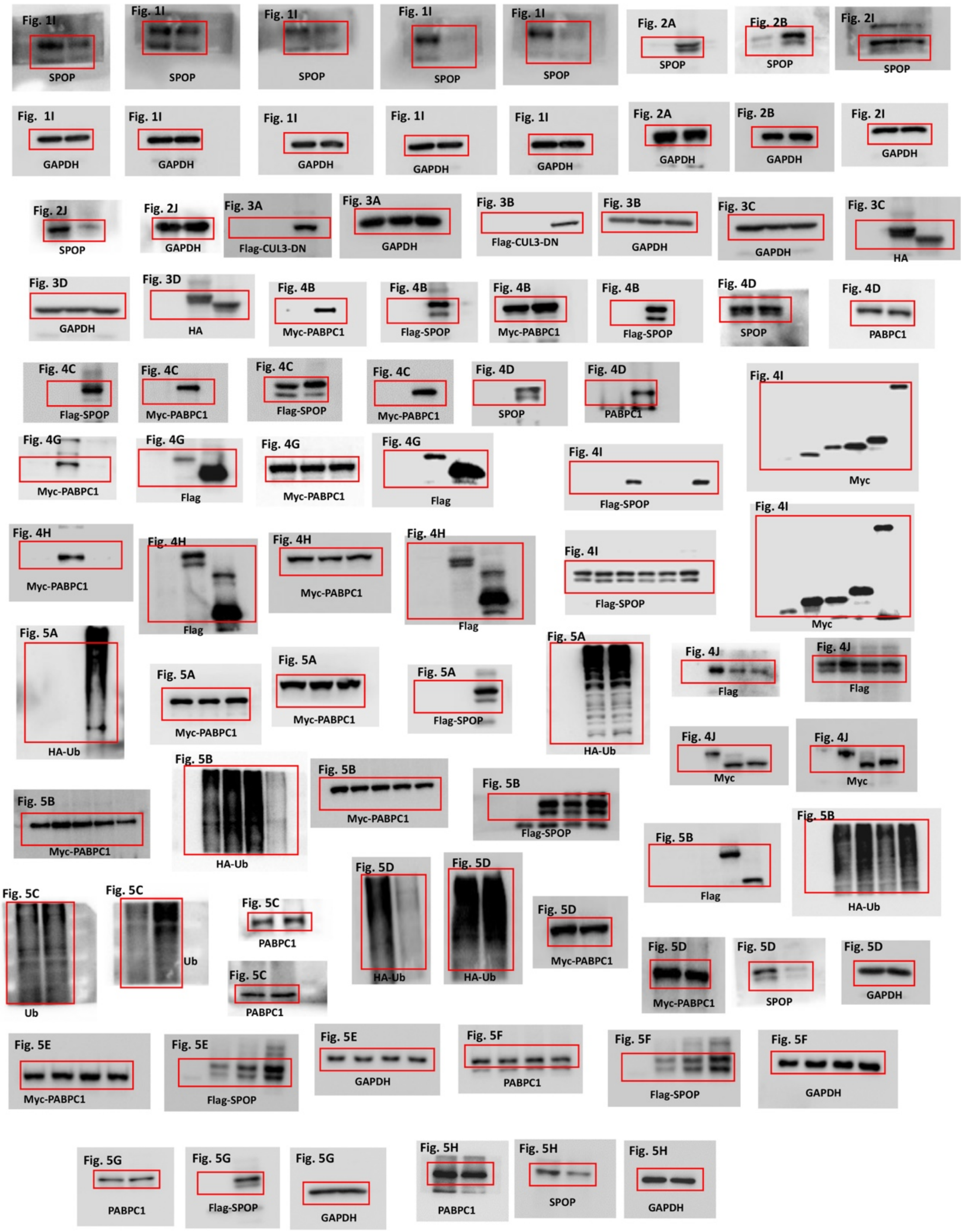

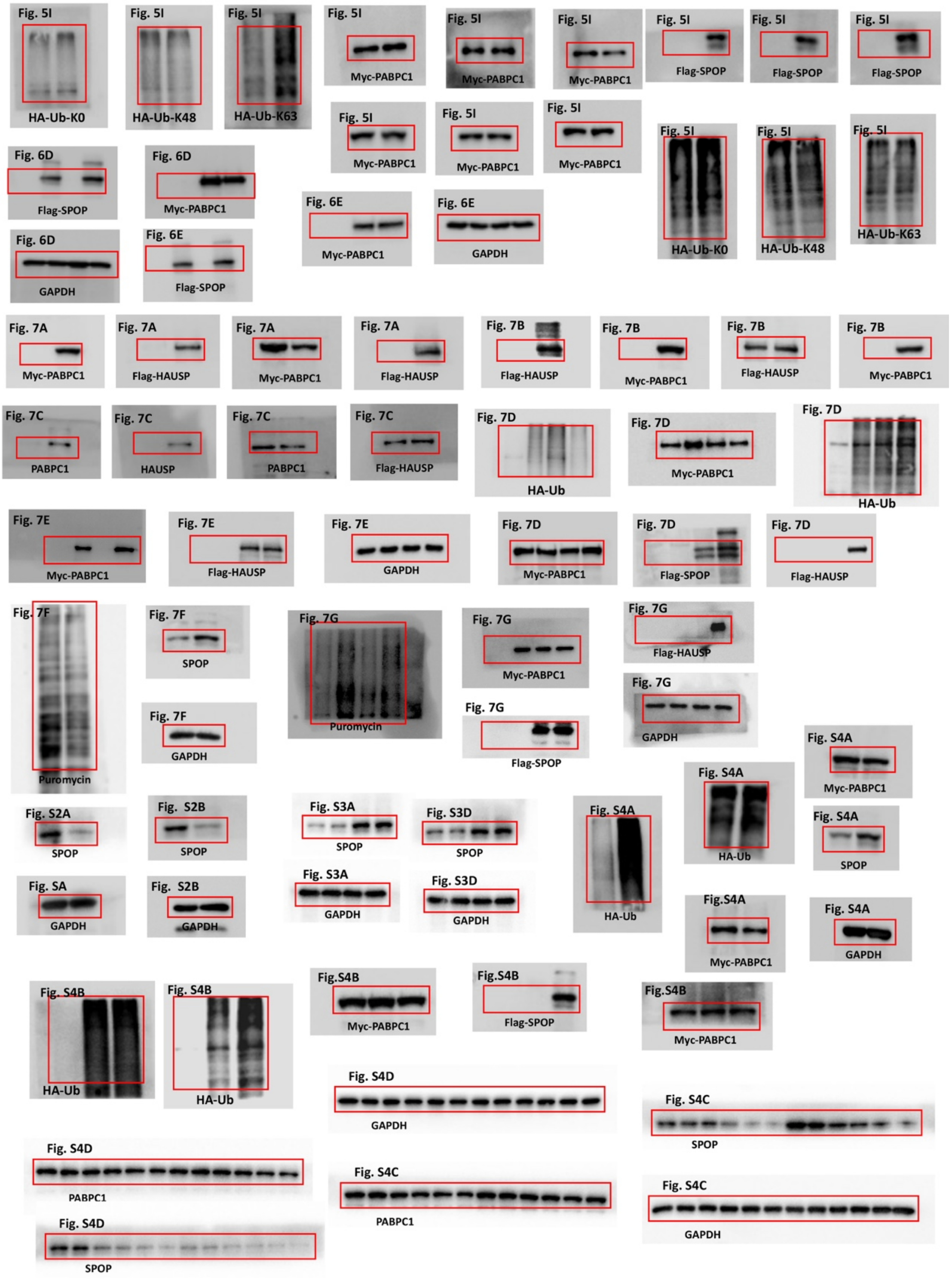
Fig. 5I
HA-Ub-K0
Fig. 5I
HA-Ub-K48
Fig. 5I
HA-Ub-K63
Fig. 5I
Myc-PABPC1
Fig. 5I
Myc-PABPC1
Fig. 5I
Myc-PABPC1
Fig. 5I
Flag-SPOP
Fig. 5I
Flag-SPOP
Fig. 5I
Flag-SPOP
Fig. 5I
Myc-PABPC1
Fig. 5I
Myc-PABPC1
Fig. 5I
Myc-PABPC1
Fig. 5I
HA-Ub-K0
Fig. 5I
HA-Ub-K48
Fig. 5I
HA-Ub-K63
Fig. 6D
Flag-SPOP
Fig. 6D
Myc-PABPC1
Fig. 6E
Myc-PABPC1
Fig. 6E
GAPDH
Fig. 6D
GAPDH
Fig. 6E
Flag-SPOP
Fig. 7A
Myc-PABPC1
Fig. 7A
Flag-HAUSP
Fig. 7A
Myc-PABPC1
Fig. 7A
Flag-HAUSP
Fig. 7B
Flag-HAUSP
Fig. 7B
Myc-PABPC1
Fig. 7B
Flag-HAUSP
Fig. 7B
Myc-PABPC1
Fig. 7C
PABPC1
Fig. 7C
HAUSP
Fig. 7C
PABPC1
Fig. 7C
Flag-HAUSP
Fig. 7D
HA-Ub
Fig. 7D
Myc-PABPC1
Fig. 7D
HA-Ub
Fig. 7E
Myc-PABPC1
Fig. 7E
Flag-HAUSP
Fig. 7E
GAPDH
Fig. 7D
Myc-PABPC1
Fig. 7D
Flag-SPOP
Fig. 7D
Flag-HAUSP
Fig. 7F
Puromycin
Fig. 7F
SPOP
Fig. 7F
GAPDH
Fig. 7G
Puromycin
Fig. 7G
Myc-PABPC1
Fig. 7G
Flag-SPOP
Fig. 7G
Flag-HAUSP
Fig. 7G
GAPDH
Fig. S4A
Myc-PABPC1
Fig. S4A
HA-Ub
Fig. S4A
SPOP
Fig. S2A
SPOP
Fig. S2B
SPOP
Fig. S3A
SPOP
Fig. S3D
SPOP
Fig. S4A
HA-Ub
Fig. SA
GAPDH
Fig. S2B
GAPDH
Fig. S3A
GAPDH
Fig. S3D
GAPDH
Fig.S4A
Myc-PABPC1
Fig. S4A
GAPDH
Fig. S4B
HA-Ub
Fig. S4B
HA-Ub
Fig.S4B
Myc-PABPC1
Fig.S4B
Flag-SPOP
Fig.S4B
Myc-PABPC1
Fig. S4D
GAPDH
Fig. S4C
SPOP
Fig. S4D
PABPC1
Fig. S4C
PABPC1
Fig. S4C
GAPDH
Fig. S4D
SPOP

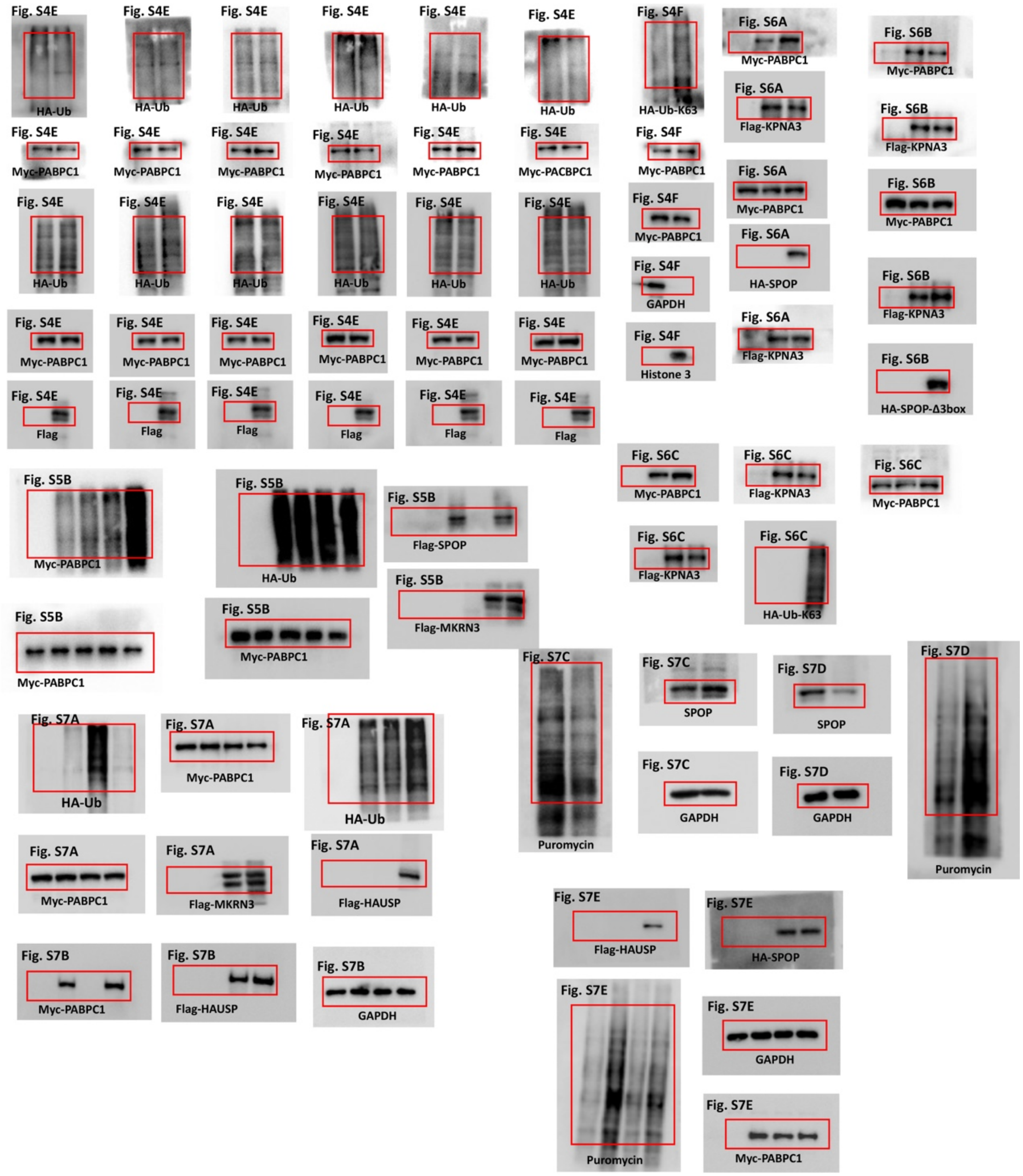
Fig. S4E
HA-Ub
Fig. S4F
HA-Ub-K63
Fig. S6A
Myc-PABPC1
Fig. S6B
Myc-PABPC1
Flag-KPNA3
Myc-PACBPC1
HA-SPOP
GAPDH
Histone 3
Flag
HA-SPOP-Δ3box
Fig. S5B
Flag-SPOP
Flag-MKRN3
Fig. S6C
Fig. S7A
Fig. S7B
Fig. S7C
Fig. S7D
Fig. S7E
SPOP
Puromycin
Flag-HAUSP
